# Screening and comparison of *in vitro*-induced resistant and clinically resistant *Stenotrophomonas maltophilia* strains for eravacycline resistance-related genes

**DOI:** 10.3389/fcimb.2025.1632701

**Published:** 2025-08-15

**Authors:** Jie Wu, Jiarong Song, Junchang Cui

**Affiliations:** Department of Respiratory Diseases, The Eighth Medical Center, Chinese People’s Liberation Army General Hospital, Beijing, China

**Keywords:** *Stenotrophomonas maltophilia*, eravacycline, *in vitro* induction, transcriptome sequencing, genome sequencing, copy number variation, efflux pump

## Abstract

**Objective:**

To identify genes related to eravacycline resistance in *Stenotrophomonas maltophilia* (*Sm*) and to provide a theoretical basis for the study of eravacycline resistance mechanisms in *Sm* and the development of new antibiotics.

**Methods:**

The study employed an integrated omics approach: (1) *In vitro* antimicrobial susceptibility profiling via broth microdilution to determine baseline MICs for eravacycline and comparator drugs; (2) Induction of resistance in clinical *Sm* isolates (WJ_4, WJ_14, WJ_18) with low eravacycline MICs through serial passage in escalating drug concentrations; (3) Transcriptome sequencing (RNA-seq) and whole-genome sequencing (WGS) of *in vitro*-induced resistant strains (WJ_4a, WJ_14a, WJ_18a) and a clinical high-MIC isolate (WJ_97); (4) Bioinformatics analyses, including differential gene expression screening (with |log2(fold change)| > 2 and FDR-adjusted p < 0.05), SNP detection via GATK, and copy number variation (CNV) quantification using CCNE-acc to identify and compare resistance-related genetic alterations.

**Results:**

Stable eravacycline-resistant strains were successfully induced, exhibiting 8- to 32-fold MIC increases. Transcriptome analysis revealed consistent upregulation of efflux pump genes (*smrA*, *smeD*, *smeE*, *smeF*) across all induced strains. CNV analysis demonstrated significant *smeDEF* locus amplification, while nonsynonymous mutations in *smeD*, *smeE*, and *smeF* occurred in WJ_14a (44 mutations) and WJ_18a (3 mutations) but not WJ_4a. The clinical strain WJ_97 showed distinct resistance architecture, including 65 upregulated nonsynonymous mutations (e.g., frameshift in *smeF*), mutations in *smeR*, *adeF*, and *vanY*-mediated target alteration, alongside a diverse resistance gene profile (41.4% inactivating enzymes, 34.4% efflux pumps). Global downregulation of cyclic AMP receptor protein (CRP) suggested synergistic multidrug resistance mechanisms.

**Conclusions:**

Eravacycline resistance in *Sm* is primarily driven by dysregulation of the *smeDEF* efflux complex, with *in vitro*-induced strains relying on CNV amplification and/or coding mutations, while clinical isolates combine these with accessory pathways. This work provides the evidence for CNV as a core resistance driver and highlights evolutionary divergence under clinical pressure, informing future antibiotic development and resistance surveillance strategies.

## 1 Introduction

*Stenotrophomonas maltophilia* (*Sm*) is a common conditionally pathogenic hospital-acquired infection pathogen, as a gram-negative bacterium that readily forms biofilms and produces virulence factors and can cause severe respiratory or bloodstream infections, especially in patients with basic lung disease or who are immunocompromised (Guerci et al., 2019; Kang et al., 2020). All-cause mortality among patients infected with *Sm* ranges from 18% to 69%, and *Sm*-attributable mortality ranges from 24% to 58% at various time points after infection (Mojica et al., 2022). The overall mortality rate of patients with pneumonia is over 60% (Kim et al., 2019). The increased isolation rate and drug resistance of *Sm* in China should not be ignored, and the clinical isolation rate of CHINET Drug Resistance Surveillance 2023 revealed *Sm* to be the 8th most common strain isolated from respiratory specimens (5.2%). And the percentage of isolated strains in CHINET Drug Resistance Surveillance 2024 exhibiting resistance to first-line drugs such as levofloxacin (LVFX), trimethoprim-sulfamethoxazole (TMP-SMZ), tigecycline, and minocycline at 8.4%, 6.5%, 5.9%, and 1.4%, respectively. This further restricts the clinical treatment options for *Sm* infections, which already has few effective therapies, making prevention and control more difficult and posing a substantial challenge to treatment.

Eravacycline, a fourth-generation tetracycline derivative, is a recently synthesized fluorocycline antibiotic that in 2018 was approved by the U.S. Food and Drug Administration (FDA) for use in intra-abdominal infections in patients 18 years of age and older, with a recommended dosage of 1 mg/kg every 12 h and an infusion duration of 1 hour (Zhanel et al., 2016). Eravacycline has also been used in some real-world studies to consolidate its anti-infective efficacy, with dual coverage for suspected carbapenem-resistant Enterobacteriaceae (CRE) infections and intolerance to other tetracyclines, among other conditions. In addition to its broad-spectrum antimicrobial activity, eravacycline is effective for infections that are resistant to tetracycline antibiotics, with favorable *in vitro* activity against *Sm*, including resistant strains (Grossman et al., 2012; Bassetti and Righi, 2014; Al Musawa et al., 2025).

Currently, there are limited drug options available to treat infections with extremely resistant strains of *Sm*, and the development of eravacycline resistance may have a serious impact on mortality of critically ill patients. However, the mechanism of resistance to eravacycline in this bacterium is unclear; therefore, studies on the mechanism of resistance to eravacycline in this bacterium are necessary.

## 2 Materials and methods

### 2.1 Experimental strains and drugs

A total of 77 unique LVFX-insensitive and/or TMP-SMZ-resistant strains of *Sm* were isolated from sputum and airway aspirate samples from June 2020 to August 2022 from the Clinical Microbiology Laboratory of the First Medical Center of the General Hospital of the People’s Liberation Army. According to the results of the minimum inhibitory concentration (MIC) measurements, three clinical eravacycline low-MIC isolates (MIC ≤ 4 mg/L) were selected, namely, one LVFX-sensitive TMP-SMZ-resistant strain (WJ_4), one LVFX-insensitive TMP-SMZ-sensitive strain (WJ_14), and one LVFX-insensitive and TMP-SMZ-resistant strain (WJ_18). One clinical isolate with a high MIC for eravacycline (16 mg/L), was identified and referred to as WJ_97. *Escherichia coli* ATCC 25922 was used as the experimental quality control strain for minocycline and TMP-SMZ, and *Pseudomonas aeruginosa* ATCC 27853 was used as the control for the other drugs. Eravacycline hydrochloride was purchased from MedChemExpress (USA); tigecycline was purchased from Shanghai Yuanye Technology & Biology Co., Ltd. (China); and minocycline hydrochloride, doxycycline, levofloxacin, moxifloxacin hydrochloride, TMP-SMZ, ceftazidime, ticarcillin, and clavulanic acid were purchased from National Institutes for Food and Drug Control (China). Mueller–Hinton agar (MHA) and Mueller-Hinton Broth (MHB) were obtained from Becton, Dickinson and Company (USA).

### 2.2 Antimicrobial susceptibility testing

The MICs of eravacycline, minocycline, tigecycline, doxycycline, levofloxacin, moxifloxacin, TMP-SMZ, ceftazidime, and ticarcillin/clavulanic acid of the insensitive *Sm* were measured *in vitro* by the microbroth dilution method in strict accordance with the standards of CLSI document M07-Ed11 (2018) (CLSI, 2018).

### 2.3 *In vitro* induction experiments

*In vitro* induction resistance culture of the experimental strains WJ_4, WJ_14 and WJ_18 was carried out by the low-concentration broth passaging induction method. The initial induction concentration of eravacycline was 1/4 the MIC of the strains, and this concentration was doubled sequentially until the concentration of eravacycline was 8 MIC. After that, stability was verified through drug-free passage testing. Control cultures without antimicrobial agents and without bacteria were also established. The eravacycline MIC value of the induced *Sm* was determined.

### 2.4 RNA extraction and transcriptome sequencing

The sensitive strains WJ_4, WJ_14 and WJ_18; the resistant strains WJ_4a, WJ_14a and WJ_18a obtained by *in vitro* induction culture; and the clinical high-MIC strain WJ_97 were inoculated on MHA solid media and cultured at 37°C for 24 hours. Total RNA was subsequently extracted from each strain individually using the TRIzol RNA Extraction Kit, and any remaining genomic DNA contamination was removed using DNase I. After quantification and quality control, the mRNAs were enriched by removing ribosomal RNA (rRNA) from the total RNA using a specific probe, followed by the addition of fragmentation buffer to randomly break the resulting mRNAs into short fragments for the construction of cDNA libraries according to a strand-specific library construction protocol. High-throughput sequencing was performed using the Illumina NovaSeq 6000 platform.

### 2.5 DNA extraction and genome sequencing

Single colonies of the sensitive strains WJ_4, WJ_14 and WJ_18; the drug-resistant strains WJ_4a, WJ_14a and WJ_18a obtained from *in vitro*-induced culture; and the high-MIC clinical strain WJ_97 were inoculated into MHA solid medium and incubated at 37°C for 24 hours, after which bacterial DNA was collected and extracted using the Bacterial DNA Extraction Kit (Magnetic Bead Method) with reference to the instruction manual. After quantification and quality control, the DNA molecules were randomly sheared into 300–400 bp fragments using physical methods, followed by end-repair, A-tailing, and ligation with paired-end adapters. The 500 bp inserts were amplified by PCR, and a paired library with a 5 kb insert size was constructed. High-throughput sequencing was performed using the Illumina NovaSeq 6000 platform.

### 2.6 Bioinformatics analysis

#### 2.6.1 Transcriptome

Low-quality sequences and adapter sequences were removed using Trimmomatic. Transcriptome sequencing reads were compared to the reference genome (NCTC10258: https://www.ncbi.nlm.nih.gov/datasets/genome/GCF_900475405.1/) using Bowtie2 software. Gene expression levels were assessed by the number of sequencing reads aligned per kilobase base length per million sequencing reads (FPKM). RNA-seq counts were modeled under a negative binomial distribution, with significance determined by an exact test (p-values adjusted for false discovery rate [FDR] via Benjamini–Hochberg). Genes with |log_2_(fold change)| > 2 and FDR-adjusted p-value (padj) < 0.05 were classified as significant.

#### 2.6.2 Genome

Low-quality sequences and adapter sequences were removed using Trimmomatic. The assembled genome was subjected to gene prediction and exhaustive annotation work, including functional resolution and taxonomic calibration, with the help of the Prokka tool. A circos plot of the genome was created using the Proksee online tool to visualize the structural features and functionality distribution in the genome in a clear and intuitive manner. GATK software was used to compare the sequencing results with the reference genome, and single nucleotide polymorphisms were identified for subsequent analysis. SNPs between different samples were compared, SNPs that differed within samples were identified, and key SNPs were labeled. The copy numbers of the efflux pump genes were estimated using CCNE-acc(https://github.com/biojiang/ccne), a component of the Carbapenemase-Encoding Gene Copy Number Estimator (CCNE) tool. We added related efflux pump genes to ccne/config/CARD_AMR_clustered.csv to enable targeted analysis. CCNE-acc assumes that read coverage for each position of the whole genome and the target gene-containing fragment follow a Poisson distribution, and it estimates the target gene copy number by this equation: copy number(CCNE-acc)_target gene_ = (λ_target gene_)/(λ_Genome)_, where λ_target gene_ and λ_Genome_ are the parameters of two Poisson distributions and are approximated by the medians of read coverages of target gene-containing fragments and the whole genome.

## 3 Results

### 3.1 Induced eravacycline-resistant strains

#### 3.1.1 *In vitro* antimicrobial susceptibility

The MIC values of eravacycline for the three strains WJ_4, WJ_14, and WJ_18 were 4 mg/L, 0.5 mg/L, and 1 mg/L, respectively, prior to the induction treatment and increased to ≥16 mg/L after *in vitro* induction with eravacycline and subsequent drug-free subculture for 10 generations (**Table 1**). The time required to induce a satisfactory resistant strain was approximately 40 days. This result indicates that we successfully and stably induced high MICs for eravacycline in three strains that originally had low MICs. To identify these changes, we named them WJ_4a, WJ_14a, and WJ_18a, respectively.

**Table 1:** The MICs of the *in vitro*-induced strains.

Strain	Pre-induction MIC (mg/L)	Post-induction MIC (mg/L)	Post-passage MIC (mg/L)
WJ_4	4	32	32
WJ_14	0.5	16	16
WJ_18	1	16	16

#### 3.1.2 Screening of differentially expressed genes

**Figure 1:**
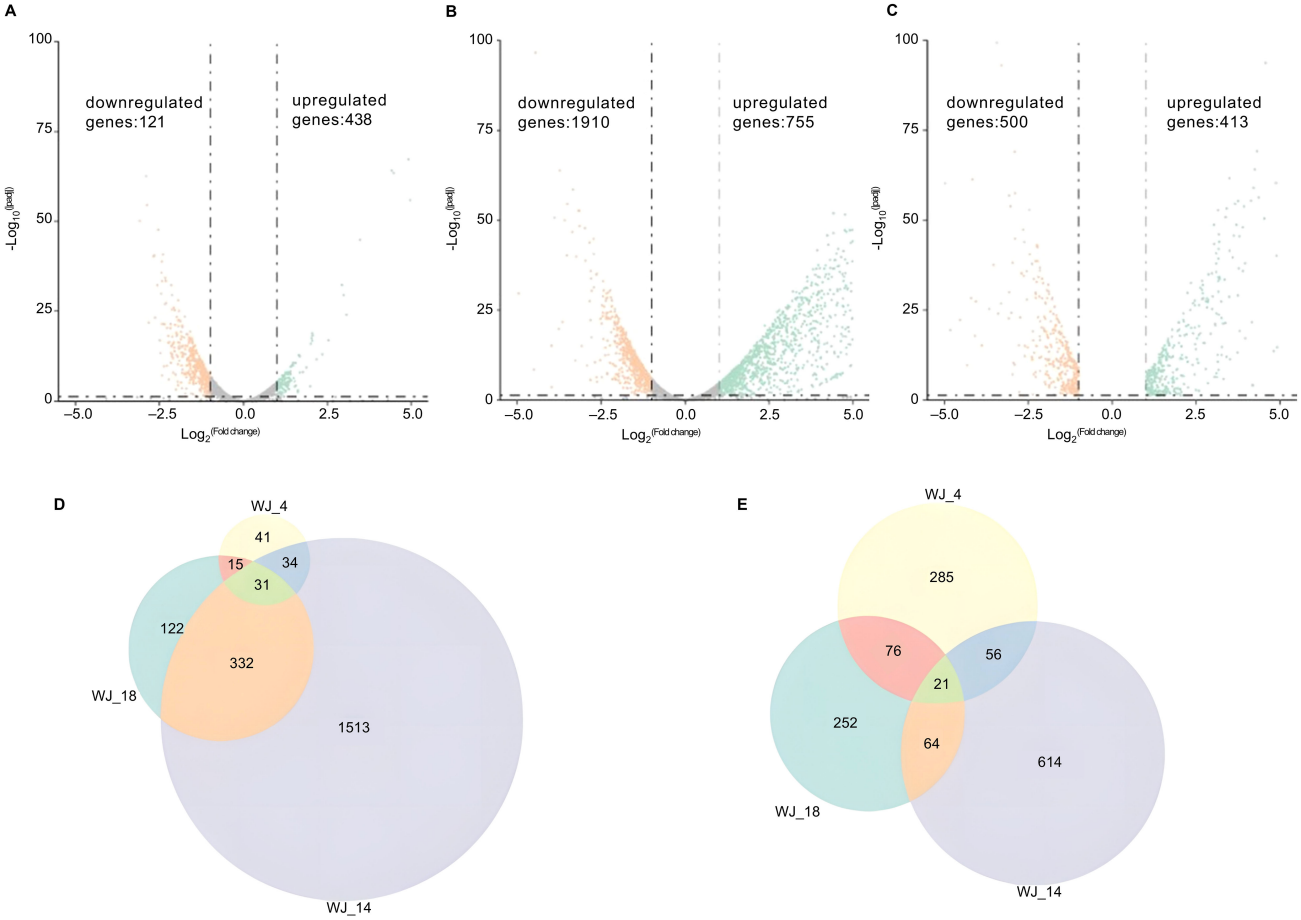
Differential gene expression analysis and identification of co-regulated genes. **(A–C)** Volcano plots of transcriptomic profiles comparing *in vitro*-induced eravacycline-resistant strains (WJ_4a, WJ_14a, WJ_18a) with their parental susceptible counterparts (WJ_4, WJ_14, WJ_18). The statistical analysis was performed by R, and the padj were caculated by an exact test. Differentially expressed genes were defined as those with |log_2_(fold change)| > 2 and padj < 0.05. **(A)** WJ_4a comparing with WJ_4: 121 upregulated (orange) and 438 downregulated genes (green). **(B)** WJ_14a comparing with WJ_14: 1,910 upregulated and 755 downregulated genes. **(C)** WJ_18a comparing with WJ_18: 500 upregulated and 413 downregulated genes. **(D)** Co-upregulated genes in WJ_4a, WJ_14a and WJ_18a(31 total). **(E)** Co-downregulated genes in WJ_4a, WJ_14a and WJ_18a (21 total).

To identify the genes that may be responsible for eravacycline resistance, we performed differential gene expression analysis of the induced eravacycline-resistant strains and original strains. In the WJ_4 group and its resistant strain WJ_4a, we identified 121 upregulated genes and 438 downregulated genes (**Figure 1A**); in the WJ_14 group and its resistant strain WJ_14a, we identified 1,910 upregulated genes and 755 downregulated genes (**Figure 1B**); in the WJ_18 group and its resistant strain WJ_18a, we identified 500 upregulated genes and 413 downregulated genes (**Figure 1C**). Despite the different origins of these three strains, all of them were induced to acquire eravacycline resistance. Therefore, the differentially expressed genes may be the factors that contributed to the development of this resistance; specifically, it is possible that genes showing the same change trend in all three groups are the main cause. Therefore, we identified the genes that were simultaneously upregulated or downregulated in all three groups. We detected a total of 31 genes whose expression was upregulated in all three groups (**Figure 1D**, **Table 2**), of which four were drug efflux transporter-related genes (*smrA*, *smeD*, *smeE* and *smeF*). We also detected a total of 21 genes whose expression was downregulated in all three groups (**Figure 1E**, **Table 3**); however, no resistance- or drug efflux transporter-related genes were found among these genes. Despite this, the global regulator cyclic AMP receptor protein (CRP) expression was co- downregulated in all three groups, suggesting that resistance may be synergistically enhanced through multidimensional mechanisms. For example, to release the inhibition of efflux pump, to promote the formation of biofilm, and to affect other drug-resistant pathways (Liu et al., 2025).

**Table 2:** Genes co-expressed up-regulated in all three groups.

Gene	Description	Fold change		
		WJ4	WJ14	WJ18
*DQN92_RS02950*	fimbrial protein	4.9	15.6	3.7
*DQN92_RS02945*	fimbrial biogenesis outer membrane usher protein	4.4	7.7	5.6
*DQN92_RS02940*	molecular chaperone	4.5	6.1	5.7
*DQN92_RS02935*	fimbrial protein	5.0	6.6	5.9
*smrA*	multidrug efflux ABC transporter SmrA	3.5	2.7	3.4
*aqpZ*	aquaporin Z	2.9	4.9	2.2
*Novel00028*	PF13954: PapC N-terminal domain	3.0	3.9	4.6
*sRNA00016*	–	5.3	6.6	6.6
*Novel00029*	–	3.1	12.6	3.9
*smeD*	multidrug efflux RND transporter periplasmic adaptor subunit SmeD	2.0	1.4	3.2
*smeE*	multidrug efflux RND transporter permease subunit SmeE	2.1	2.4	3.5
*smeF*	multidrug efflux RND transporter outer membrane subunit SmeF	2.0	2.7	3.0
*agp*	bifunctional glucose-1-phosphatase/inositol phosphatase	2.0	4.5	3.7
*DQN92_RS09725*	MliC family protein	1.7	1.6	1.5
*DQN92_RS14335*	hypothetical protein	2.2	13.5	2.9
*DQN92_RS09660*	hypothetical protein	1.5	11.3	2.7
*DQN92_RS13030*	hypothetical protein	1.8	15.9	2.5
*DQN92_RS09655*	hypothetical protein	1.3	11.5	1.5
*DQN92_RS16330*	hypothetical protein	1.3	13.8	1.3
*DQN92_RS00090*	hypothetical protein	1.2	12.7	12.8
*pgaC*	poly-beta-1%2C6-N-acetyl-D-glucosamine synthase	1.5	1.7	1.6
*DQN92_RS19930*	methyl-accepting chemotaxis protein	1.3	3.4	3.2
*DQN92_RS00080*	hypothetical protein	1.4	12.4	12.3
*pgaA*	poly-beta-1%2C6 N-acetyl-D-glucosamine export porin PgaA	1.3	2.5	1.6
*DQN92_RS07315*	hypothetical protein	1.3	11.3	1.7
*Novel00248*	PF16576: Barrel-sandwich domain of CusB or HlyD membrane-fusion	1.1	1.7	2.3
*pgaD*	poly-beta-1%2C6-N-acetyl-D-glucosamine biosynthesis protein PgaD	1.3	2.2	1.6
*DQN92_RS04425*	tRNA-Asp	1.0	1.0	1.4
*DQN92_RS13000*	hypothetical protein	1.0	11.2	1.1
*DQN92_RS20945*	hypothetical protein	1.6	12.1	11.9
*DQN92_RS09595*	heavy-metal-associated domain-containing protein	1.2	12.4	6.5

**Table 3:** Genes co-expressed down-regulated in all three groups.

Gene	Description	Fold change		
		WJ4	WJ14	WJ18
*DQN92_RS03730*	S8 family peptidase	-4.3	-1.3	-3.7
*DQN92_RS01315*	CsbD family protein	-2.9	-2.3	-5.1
*DQN92_RS00200*	hypothetical protein	-2.3	-4.2	-1.3
*crp*	cAMP-activated global transcriptional regulator CRP	-2.3	-3.3	-3.4
*DQN92_RS02840*	glycosyl hydrolase family 18 protein	-2.3	-2.5	-2.1
*DQN92_RS17470*	TonB-dependent receptor	-2.3	-3.6	-2.2
*DQN92_RS00905*	DUF1328 domain-containing protein	-2.0	-2.8	-4.2
*DQN92_RS03040*	hypothetical protein	-1.8	-3.1	-1.7
*Novel00032*	–	-2.1	-3.5	-2.1
*DQN92_RS00205*	DUF4785 family protein	-2.5	-2.3	-1.9
*DQN92_RS11230*	glucose 1-dehydrogenase	-2.1	-2.2	-2.0
*glgX*	glycogen debranching protein GlgX	-1.3	-2.4	-1.6
*DQN92_RS19360*	DNA breaking-rejoining protein	-1.4	-5.4	-2.2
*DQN92_RS00125*	hypothetical protein	-1.4	-1.6	-1.9
*DQN92_RS19335*	sorbosone dehydrogenase family protein	-1.1	-2.0	-3.0
*DQN92_RS17395*	NAD(P)-dependent alcohol dehydrogenase	-1.1	-4.2	-2.2
*DQN92_RS19365*	SCO family protein	-1.3	-1.8	-4.2
*Novel00244*	–	-1.2	-1.8	-2.8
*DQN92_RS20100*	hypothetical protein	-1.2	-1.9	-1.4
*DQN92_RS00495*	DUF58 domain-containing protein	-1.5	-2.8	-4.1
*ribD*	5-amino-6-(5-phosphoribosylamino) uracil reductase RibD	-1.1	-2.9	-6.2

#### 3.1.3 Genome-wide sequence comparison of genes related to drug efflux transporters

To investigate the gene mutations associated with eravacycline resistance, we sequenced the genomes of the three resistant strains and the corresponding sensitive strains and focused on genes significantly upregulated in the resistant strains at the transcriptome level, especially drug efflux transporter-related genes. Interestingly, SNPs at the genome level were found only in the three significantly upregulated drug efflux transporter-related genes; the remaining genes did not exhibit mutations. These three genes, *smeD*, *smeE* and *smeF*, were identified as having 403 single-nucleotide polymorphisms in WJ_14a compared with the sensitive control WJ_14, including 359 synonymous and 44 nonsynonymous mutations, the latter of which may have a greater impact on protein structure. Specifically, the *smeD* gene had 8 nonsynonymous mutations, *smeE* had 16, and smeF had 20. Twenty-five single-nucleotide variants were identified in WJ_18a, including 22 synonymous and 3 nonsynonymous mutations, with *smeD*, *smeE*, and *smeF* having one nonsynonymous mutation each. Notably, no SNPs were found in WJ_4a (**Supplementary Tables S1**, **S2**). Moreover, no screened SNPs were shared between WJ_14a and WJ_18a.

#### 3.1.4 Copy number variation of the *smeDEF* Efflux locus in resistant strains

To elucidate whether genomic amplification contributes to eravacycline resistance, we quantified the copy number (CN) of the *smeDEF* efflux operon in induced-resistant strains versus their susceptible progenitors using the CCNE-acc algorithm. Significant CN amplification of *smeDEF* was observed in all resistant strains (*P* < 0.001, paired t-test), with distinct magnitudes across isolates (**Table 4**): WJ_4a exhibited a 4.2-fold increase, WJ_14a showed a 2.4-fold increase, WJ_18a displayed a 7.2-fold increase. This amplification aligns with: (1) Transcriptional upregulation of *smeDEF*; (2) the absence of nonsynonymous SNPs in WJ_4a, suggesting CNV-driven overexpression as a primary resistance mechanism in this strain; (3) the heterogeneity of resistance evolution pathways, where WJ_14a and WJ_18a combined CNV with coding mutations.

**Table 4:** Copy number variation of the smeDEF efflux pump locus in susceptible vs. *in vitro*-induced eravacycline-resistant strains.

Strain Pair	Sensitive Strain (CN ± SD)	Resistant Strain (CN ± SD)
WJ_4 vs WJ_4a	1.01 ± 0.46	4.27 ± 1.17
WJ_14 vs WJ_14a	1.05 ± 0.31	2.54 ± 2.01
WJ_18 vs WJ_18a	1.21 ± 0.41	8.75 ± 0.53

#### 3.1.5 Expanded analysis of regulatory and accessory gene SNPs

In addition to resistance genes, we studied the following SNPs. (1) Regulatory regions (Promoters/UTRs): No mutations were found in conserved regulatory motifs (e.g., *smeT*-binding sites) within 1.5 kb upstream of *smeDEF*. (2) Accessory genes (Transcriptional Regulators): A synonymous mutation in *smeT* (WJ_14a: c.213G>A) showed no impact on *smeDEF* promoter activity.

As to the potential SNPs in CRP binding sites. *Sm* exhibits distinct regulatory mechanisms compared to model organisms like *E. coli*. No observed mutations: Our whole-genome SNP analysis (Snippy pipeline) of resistant vs. susceptible strains revealed no nonsynonymous SNPs or regulatory variants in predicted CRP binding motifs upstream of efflux pump genes (e.g., *smeDEF*) or other CRP-associated loci (cAMP metabolism genes).

### 3.2 Clinical high-MIC strains

#### 3.2.1 *In vitro* antimicrobial susceptibility

WJ_97 is resistant to minocycline, levofloxacin, and TMP-SMZ, has intermediate resistance to ceftazidime, and has MIC_90_ values of 8 mg/L for tigecycline, 64 mg/L for doxycycline, 16 mg/L for eravacycline, 4 mg/L for moxifloxacin, and 64/2 mg/L for ticarcillin/clavulanic acid.

#### 3.2.2 Basic genomic characteristics

**Figure 2:**
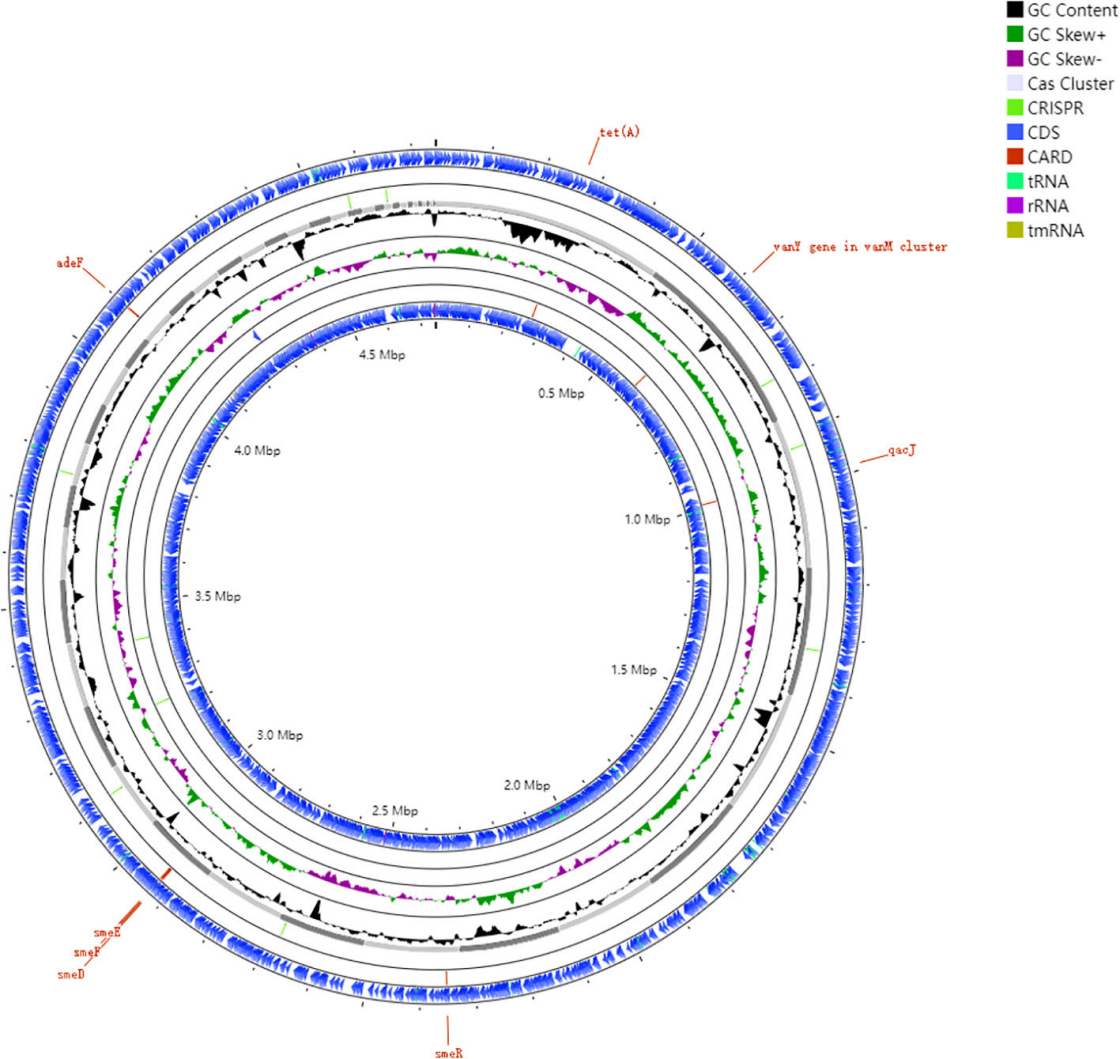
Genomic architecture of the clinical resistant strain WJ_97. Circos plot annotated against reference genome NCTC10258. Ring 1and Ring 6: CDS in forward and reverse strands; Ring 2 and Ring 5: Key resistance gene loci (*smeD*, *smeE*, *smeF*, *smeR*, *adeF*, *tet(A)*, *qacJ*, *vanY*); Ring 3: plot of GC content; Ring 4: GC skew plot; Ring 7: sequence ruler.

We performed an exhaustive annotation of the WJ_97 genome after completing its assembly. This included gene annotation using Prokka, annotation of CRISPR sequences, and annotation of CARD resistance genes. These annotation efforts provided a comprehensive and in-depth understanding of the genes in the WJ_97 genome and their functions, confirmed the presence of CRISPR sequences, and reveal possible drug resistance genes. WJ_97 possesses eight genes that are associated with drug resistance. Specifically, the efflux pump -associated genes carried by WJ_97 include *smeD*, *smeE*, *smeF*, *smeR*, and *adeF* in the RND family; *tet(A)* in the major facilitator superfamily (MFS); qacJ in the SMR family; and *vanY*, which is associated with structural changes in antibiotic target sites. **Figure 2** shows the ring structure of the WJ_97 genome. We labeled important features such as genes, GC content, CDS regions, and resistance genes in detail to better represent the genome architecture. The scale on the genome ring clearly indicates the size of the genome, and the rich labeling of genomic features in the figure further deepens our understanding of the WJ_97 genome. This figure not only displays the structural features of the WJ_97 genome but also provides a valuable reference for exploring its biological properties and potential drug resistance. Through this comprehensive analysis of the WJ_97 genome, we were able to gain a deeper understanding of its biological mechanisms and functions, which provided an important direction for further research.

#### 3.2.3 Carried resistance genes

**Figure 3:**
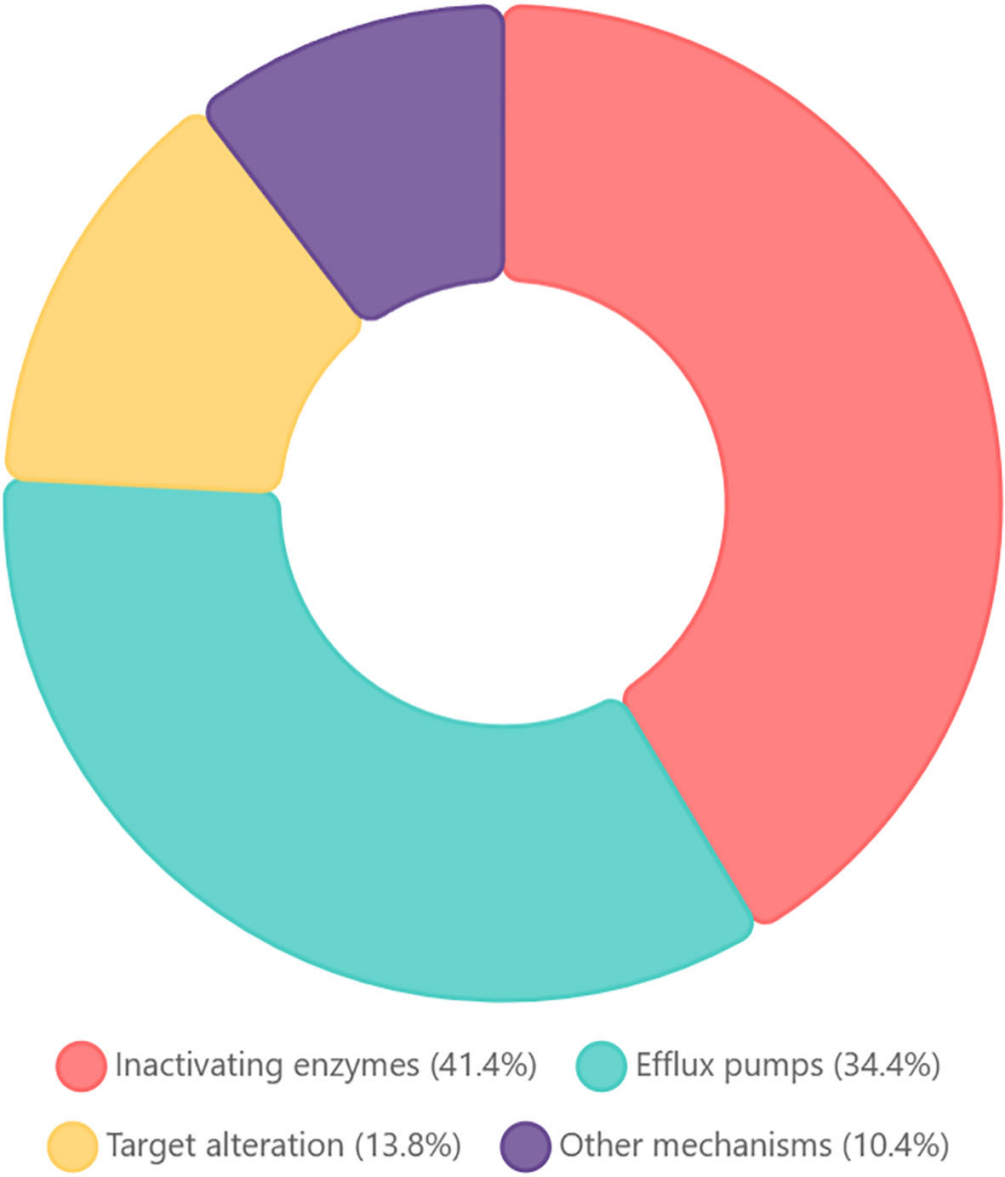
Functional classification of antibiotic resistance genes in *Stenotrophomonas maltophilia* WJ_97. The chart showing the distribution of resistance mechanisms among CARD-annotated genes: Inactivating enzymes (41.4%): enzymatic modification/degradation; efflux pumps (34.4%): transporter-mediated extrusion; target alteration (13.8%): modified antibiotic binding sites; other mechanisms (10.4%): reduced permeability, biofilm formation, and undefined pathways.

We compared the predicted protein sequences of all the genes derived from the CARD database and analyzed the drug resistance genes in the genomes of the sequenced strains (**Figure 3**). We categorized these genes according to the mechanism of resistance and found that the highest percentage of them were genes encoding antibiotic-inactivating enzymes (41.4%). Efflux pump accounted for 34.4%, target alteration accounted for 13.8% and other mechanisms accounted for 10.4%, such as permeability reduction, biofilm formation and undefined pathway.

#### 3.2.4 Comparison of transcriptome and genome sequences between the clinical high-MIC strain and the standard strain

The WJ_97 genome was compared with the reference genome NCTC10258. NCTC 10258, as an international standard strain, possessed genetic stability and publicly available antibiotic resistance phenotype data, providing a baseline reference for understanding the acquisition mechanisms of clinical antibiotic-resistant strains. 549 SNPs were found in the coexpressed up-regulated genes and 28 SNPs were found in the coexpressed down-regulated genes. Sixty-five nonsynonymous mutations were found in the SNPs of the upregulated genes. We focused on the SNPs in *smeD*, *smeE*, and *smeF*, which are related to the resistance of the mutant strain to eravacycline, among which 16 nonsynonymous mutations were found in *smeD*, 28 nonsynonymous mutations were found in *smeE*, and 21 nonsynonymous mutations were found in *smeF*. In addition, there were 4 single-base deletion mutations in *smeF*. As deletion mutations that are not multiples of 3 result in amino acid frameshifts, thus having a greater impact on protein function, we believe that these mutations in the *smeF* protein in WJ_97 may be responsible for the emergence of resistance, although this needs to be verified by further experiments. The remaining 480 SNPs were synonymous mutations. Among the 28 SNPs in the coexpressed down-regulated genes, only two nonsynonymous mutations were found in the CRP gene, while the rest were synonymous mutations (**Supplementary Table S3**).

#### 3.2.5 Comparison of genome sequences between the clinical high-MIC strain and *in vitro*-induced strains

A comparison of all resistance genes in WJ_97 with those in the mutant strains WJ4a, WJ14a, and WJ18a revealed a total of 313 SNPs. These variants included 8 deletions, 48 nonsynonymous mutations (21 in *smeR*, 7 in *smeD*, 8 in *smeE*, 6 in *smeF*, and 6 in *adeF*), 256 synonymous mutations, and 1 termination mutation (in the *smeE* gene) (**Supplementary Table S4**).

## 4 Discussion

### 4.1 Significance of induced mutation and clinical drug-resistant strains research

Both the induced mutant and the clinically resistant strains achieved eravacycline resistance through the aberrant expression of efflux pump genes, but the mechanism of resistance in the clinical strains is more complex and involves multigene synergism and the inactivation of enzymes. This suggests that the clinically resistant strains may have accumulated a wider range of adaptive mutations under the pressure of long-term antibiotic selection, whereas the *in vitro* induction model is more likely to result in a single resistance mechanism. (**Table 5**).

**Table 5:** Comparison of the induced mutant strains and the clinically resistant strain.

Comparative dimension	Induced mutant strains (WJ4_a/WJ14_a/WJ18_a)	Clinically resistant strain (WJ_97)
Core resistance mechanism	*smeDEF* efflux pump dysregulation	*smeDEF/adeF* efflux dysregulation + *vanY*-mediated target alteration
Genetic alterations	CNV amplification (2.4-7.2×) Nonsynonymous SNPs (WJ_14a:44; WJ_18a:3)	65 nonsynonymous SNPs in *smeDEF* Frameshift mutation in *smeF*
Resistance gene profile	RND family (*smeDEF*) ABC family (*smrA*)	RND family (*smeDEF/adeF*) MFS (*tetA*) SMR (*qacJ*) Target alteration (*vanY*)
Regulatory adaptation	Global CRP downregulation (no regulatory mutations)	CRP downregulation + nonsynonymous mutations (2 SNPs)

### 4.2 Characterization of *Sm* resistance mechanisms to eravacycline

In this study, transcriptome and whole-genome sequencing revealed that eravacycline resistance in *Sm* is primarily driven by genomic amplification and/or mutation of efflux pump genes.

#### 4.2.1 CNV as a primary driver of efflux pump amplification

There were synergistic activations of RND family pump *smeDEF* in the induced strains: Overexpression of *smeD* (1.4-3.2-fold upregulation), *smeE* (2.1-3.5-fold), and *smeF* (2.0-3.0-fold) was positively correlated with elevated MIC values. This finding is consistent with previous models of minocycline and tigecycline resistance (Chang et al., 2015; Cornely OA et al., 2018), further supporting the centrality of the RND family of pumps in tetracycline efflux. Our study provides the first evidence that genomic amplification of the *smeDEF* operon is a core mechanism conferring eravacycline resistance in *Sm*. Key findings support this: The amplification of *smeDEF* directly correlated with eravacycline MIC elevation, indicating a gene dosage effect where increased pump density enhances drug efflux capacity (Yaghoubi et al., 2022; Nicoloff et al., 2024). WJ_4a achieved high-level resistance (MIC=32 mg/L) without coding mutations in *smeDEF*, demonstrating that CNV alone can drive clinically relevant resistance. This challenges mutation-centric models and aligns with preadaption of intrinsic resistance genes under antibiotic pressure (García-León et al., 2014). Heterogeneity in CNV magnitude (e.g., WJ_18a: 7.2-fold vs. WJ_14a: 2.4-fold) reflects genetic background-dependent adaptation. Strains with lower CNV gains compensated via coding mutations (e.g., 44 nonsynonymous SNPs in WJ_14a), while WJ_18a combined moderate CNV with minimal mutations, indicating functional redundancy in resistance optimization (Andersson and Hughes, 2010).

CNV-mediated resistance may be reversible upon antibiotic withdrawal due to potential gene copy number instability, informing “drug holiday” strategies to restore baseline susceptibility—unlike stable SNP-based resistance (Nicoloff et al., 2024).

#### 4.2.2 CRP downregulation with intact regulatory elements drives efflux dysregulation

Based on the results, we analyzed regulatory and auxiliary factors influencing eravacycline resistance. CRP downregulation was consistently observed across all induced-resistant strains. CRP is a pivotal transcriptional regulator in bacterial antimicrobial resistance (AMR), modulating resistance through diverse pathways. CRP-cAMP complexes bind DNA to influence gene expression, enhancing resistance by altering membrane permeability, regulating efflux systems, and reducing antibiotic uptake. Additionally, CRP represses SOS response pathways, increasing persistence to β-lactams. These mechanisms underscore CRP’s role in fine-tuning bacterial AMR profiles in response to environmental stresses (Liu et al., 2025). In addition to affecting the efflux pump, its effect on biofilm formation is also worth considering. We found that the expression of the *pga* operon (**Table 2**), which affects biofilm formation, was elevated in the *in vitro* induced strains (Kishii et al., 2020; Lin et al., 2020). It is necessary to further clarify in subsequent studies whether the drug resistance phenotype of the *in vitro* induced strain is related to biofilm formation.

Regarding auxiliary factors, no mutations were detected in promoter regions (e.g., smeT binding sites) or CRP-binding motifs, suggesting that resistance arises primarily from transcriptional dysregulation, genomic amplifications (CNVs) and resistance gene SNPs rather than sequence-specific alterations in regulatory elements. This emphasizes the dominance of post-transcriptional and structural changes in efflux pump genes (e.g., smeDEF) over direct promoter-driven adaptations (Andersson and Hughes, 2010).

#### 4.2.3 Integrative resistance architecture in clinical isolate

Based on the results, the integrative resistance architecture in clinical isolate WJ_97 involves synergistic mechanisms, dominated by core efflux dysregulation through mutations in the *smeDEF* RND efflux complex (including a critical frameshift mutation in *smeF* predicted to alter substrate efflux) and dysregulation of *adeF*, which aligns with findings in induced strains and confirms *smeDEF*’s centrality. This is complemented by accessory mechanisms such as target alteration via *vanY* and inactivating enzymes (accounting for 41.4% of resistance genes), mechanisms largely absent in induced strains, alongside evolutionary divergence characterized by *smeR* mutations (21 nonsynonymous SNPs) and additional resistance genes like *tet(A)* and *qacJ*, reflecting adaptation under complex clinical antibiotic pressure. Ultimately, this multi-layered architecture demonstrates greater complexity than *in vitro*-induced resistance.

### 4.3 Comparison and insight into cross-species resistance mechanisms

Unlike in *A. baumannii* and *Klebsiella pneumoniae*, this study found that any known eravacycline-associated resistance genes (such as *adeB* and *acrAB-TolC*) were absent (Abdallah et al., 2015; Xu et al., 2022),suggesting that *Sm* may circumvent the traditional resistance pathways through unique genetic elements. This finding has dual significance, as follows. (1) Clinical treatment strategy: the inhibitory effect of existing efflux pump inhibitors on smeDEF still needs to be validated. The combination of drugs (e.g., eravacycline + pump inhibitor) may become an important direction for delaying drug resistance. (2) Monitoring of resistance evolution: plasmid-mediated *tet(Y)* genes can spread horizontally in other strains (Wang et al., 2021), whereas the genes for *Sm* resistance to eravacycline are mainly located on the chromosome, implying that resistance may spread through vertical evolution rather than horizontal transfer. A long-term surveillance system is needed to assess the epidemiologic impact of this discrepancy.

Although this study initially revealed the central role of the *smeDEF* in eravacycline resistance, the following limitations remain. (1) Functional validation is missing: the contribution of each efflux pump needs to be clarified via knockout/backfill experiments. (2) Unclear regulatory network: The mutation frequency of regulatory factors and their association with phenotypes need to be further analyzed. (3) Insufficient clinical samples: the cohort of drug-resistant strains needs to be expanded to confirm the prevalence of mutation sites. Future studies should integrate multi-omics data to systematically analyze the molecular regulatory network of *Sm* resistance and provide targets for the development of novel inhibitors.

## Data Availability

The raw sequencing data were deposited in the NCBI SRA under accession numbers PRJNA1273168 and PRJNA1273497.
