# Supplementary material for: Screening and comparison of in vitro-induced resistant and clinically resistant Stenotrophomonas maltophilia strains for eravacycline resistance-related genes
Source: Front Cell Infect Microbiol. 2025 Aug 15;15:1632701. doi: 10.3389/fcimb.2025.1632701 (PMC12394494; doi:10.3389/fcimb.2025.1632701)
Supplement: Supplementary file 1 [file Table1.docx]

Supplementary Material

# Supplementary Tables

Table S1 SNPs that WJ_14a has compared to WJ_14

| Chromosomes | Position | Type of SNP | Gene | Mutation mode | Amino acid mutations |
| --- | --- | --- | --- | --- | --- |
| NZ_LS483377.1 | 3825606 | syn | smeD | G<->A | A1149:2A |
| NZ_LS483377.1 | 3825607 | nonsyn | smeD | G<->T | A1148:1D |
| NZ_LS483377.1 | 3825609 | syn | smeD | C<->G | A1146:2A |
| NZ_LS483377.1 | 3825612 | syn | smeD | T<->C | A1143:2A |
| NZ_LS483377.1 | 3825627 | syn | smeD | A<->T | A1128:2A |
| NZ_LS483377.1 | 3825630 | syn | smeD | A<->G | A1125:2A |
| NZ_LS483377.1 | 3825631 | nonsyn | smeD | G<->T | A1124:1D |
| NZ_LS483377.1 | 3825639 | syn | smeD | C<->T | E1116:2E |
| NZ_LS483377.1 | 3825687 | syn | smeD | G<->A | V1068:2V |
| NZ_LS483377.1 | 3825702 | syn | smeD | G<->A | G1053:2G |
| NZ_LS483377.1 | 3825714 | syn | smeD | A<->G | G1041:2G |
| NZ_LS483377.1 | 3825846 | syn | smeD | T<->C | V909:2V |
| NZ_LS483377.1 | 3825888 | syn | smeD | G<->A | R867:2R |
| NZ_LS483377.1 | 3825897 | syn | smeD | G<->C | T858:2T |
| NZ_LS483377.1 | 3825898 | nonsyn | smeD | G<->A | T857:1I |
| NZ_LS483377.1 | 3825944 | syn | smeD | G<->A | L811:0L |
| NZ_LS483377.1 | 3825962 | nonsyn | smeD | C<->T | A793:0T |
| NZ_LS483377.1 | 3825963 | syn | smeD | T<->C | P792:2P |
| NZ_LS483377.1 | 3825975 | syn | smeD | C<->G | V780:2V |
| NZ_LS483377.1 | 3825981 | syn | smeD | A<->G | S774:2S |
| NZ_LS483377.1 | 3826047 | syn | smeD | C<->G | T708:2T |
| NZ_LS483377.1 | 3826101 | syn | smeD | C<->T | E654:2E |
| NZ_LS483377.1 | 3826107 | syn | smeD | C<->G | S648:2S |
| NZ_LS483377.1 | 3826128 | syn | smeD | G<->A | Y627:2Y |
| NZ_LS483377.1 | 3826155 | syn | smeD | G<->A | A600:2A |
| NZ_LS483377.1 | 3826200 | syn | smeD | A<->G | V555:2V |
| NZ_LS483377.1 | 3826326 | syn | smeD | C<->G | V429:2V |
| NZ_LS483377.1 | 3826488 | Syn | smeD | G<->C | L267:2L |
| NZ_LS483377.1 | 3826614 | syn | smeD | G<->A | G141:2G |
| NZ_LS483377.1 | 3826623 | syn | smeD | C<->T | E132:2E |
| NZ_LS483377.1 | 3826680 | syn | smeD | T<->C | Q75:2Q |
| NZ_LS483377.1 | 3822501 | syn | smeE | A<->G | G3057:2G |
| NZ_LS483377.1 | 3822573 | syn | smeE | A<->G | G2985:2G |
| NZ_LS483377.1 | 3822615 | syn | smeE | C<->G | L2943:2L |
| NZ_LS483377.1 | 3822636 | syn | smeE | A<->G | R2922:2R |
| NZ_LS483377.1 | 3822657 | syn | smeE | A<->G | H2901:2H |
| NZ_LS483377.1 | 3822669 | syn | smeE | T<->C | E2889:2E |
| NZ_LS483377.1 | 3822680 | nonsyn | smeE | C<->T | G2878:0S |
| NZ_LS483377.1 | 3822681 | syn | smeE | T<->G | A2877:2A |
| NZ_LS483377.1 | 3822690 | syn | smeE | C<->T | E2868:2E |
| NZ_LS483377.1 | 3822699 | syn | smeE | C<->T | E2859:2E |
| NZ_LS483377.1 | 3822714 | syn | smeE | C<->G | V2844:2V |
| NZ_LS483377.1 | 3822774 | syn | smeE | G<->A | Y2784:2Y |
| NZ_LS483377.1 | 3822780 | syn | smeE | A<->G | D2778:2D |
| NZ_LS483377.1 | 3822786 | syn | smeE | T<->C | E2772:2E |
| NZ_LS483377.1 | 3822804 | syn | smeE | A<->G | N2754:2N |
| NZ_LS483377.1 | 3822828 | syn | smeE | G<->A | G2730:2G |
| NZ_LS483377.1 | 3822852 | syn | smeE | T<->C | A2706:2A |
| NZ_LS483377.1 | 3822861 | syn | smeE | C<->T | V2697:2V |
| NZ_LS483377.1 | 3822876 | syn | smeE | G<->A | Y2682:2Y |
| NZ_LS483377.1 | 3822900 | syn | smeE | T<->C | V2658:2V |
| NZ_LS483377.1 | 3822915 | syn | smeE | G<->C | L2643:2L |
| NZ_LS483377.1 | 3822945 | syn | smeE | A<->G | A2613:2A |
| NZ_LS483377.1 | 3822954 | syn | smeE | C<->T | E2604:2E |
| NZ_LS483377.1 | 3822978 | syn | smeE | T<->C | E2580:2E |
| NZ_LS483377.1 | 3822999 | syn | smeE | C<->A | L2559:2L |
| NZ_LS483377.1 | 3823017 | syn | smeE | T<->C | E2541:2E |
| NZ_LS483377.1 | 3823038 | syn | smeE | A<->G | G2520:2G |
| NZ_LS483377.1 | 3823074 | syn | smeE | T<->C | E2484:2E |
| NZ_LS483377.1 | 3823080 | syn | smeE | G<->C | A2478:2A |
| NZ_LS483377.1 | 3823101 | syn | smeE | T<->C | E2457:2E |
| NZ_LS483377.1 | 3823143 | syn | smeE | G<->T | A2415:2A |
| NZ_LS483377.1 | 3823161 | syn | smeE | T<->C | E2397:2E |
| NZ_LS483377.1 | 3823164 | syn | smeE | A<->G | G2394:2G |
| NZ_LS483377.1 | 3823191 | syn | smeE | A<->G | D2367:2D |
| NZ_LS483377.1 | 3823206 | syn | smeE | C<->G | V2352:2V |
| NZ_LS483377.1 | 3823242 | syn | smeE | A<->G | R2316:2R |
| NZ_LS483377.1 | 3823272 | syn | smeE | G<->A | D2286:2D |
| NZ_LS483377.1 | 3823296 | syn | smeE | G<->A | A2262:2A |
| NZ_LS483377.1 | 3823434 | syn | smeE | G<->C | L2124:2L |
| NZ_LS483377.1 | 3823455 | syn | smeE | C<->T | A2103:2A |
| NZ_LS483377.1 | 3823479 | syn | smeE | C<->T | Q2079:2Q |
| NZ_LS483377.1 | 3823524 | syn | smeE | G<->A | G2034:2G |
| NZ_LS483377.1 | 3823536 | syn | smeE | G<->A | I2022:2I |
| NZ_LS483377.1 | 3823557 | syn | smeE | G<->A | F2001:2F |
| NZ_LS483377.1 | 3823599 | syn | smeE | G<->A | R1959:2R |
| NZ_LS483377.1 | 3823602 | syn | smeE | T<->A | G1956:2G |
| NZ_LS483377.1 | 3823628 | nonsyn | smeE | T<->C | N1930:0D |
| NZ_LS483377.1 | 3823713 | syn | smeE | C<->G | S1845:2S |
| NZ_LS483377.1 | 3823725 | syn | smeE | G<->A | D1833:2D |
| NZ_LS483377.1 | 3823761 | syn | smeE | T<->C | E1797:2E |
| NZ_LS483377.1 | 3823766 | syn | smeE | G<->A | L1792:0L |
| NZ_LS483377.1 | 3823788 | syn | smeE | A<->G | R1770:2R |
| NZ_LS483377.1 | 3823791 | syn | smeE | T<->C | E1767:2E |
| NZ_LS483377.1 | 3823803 | syn | smeE | A<->G | G1755:2G |
| NZ_LS483377.1 | 3823806 | syn | smeE | C<->G | V1752:2V |
| NZ_LS483377.1 | 3823832 | syn | smeE | A<->G | L1726:0L |
| NZ_LS483377.1 | 3823860 | syn | smeE | T<->C | S1698:2S |
| NZ_LS483377.1 | 3823883 | syn | smeE | A<->G | L1675:0L |
| NZ_LS483377.1 | 3823905 | syn | smeE | C<->T | A1653:2A |
| NZ_LS483377.1 | 3823935 | syn | smeE | G<->A | R1623:2R |
| NZ_LS483377.1 | 3823956 | syn | smeE | G<->A | G1602:2G |
| NZ_LS483377.1 | 3823968 | syn | smeE | G<->A | S1590:2S |
| NZ_LS483377.1 | 3823989 | syn | smeE | T<->G | G1569:2G |
| NZ_LS483377.1 | 3824001 | syn | smeE | G<->A | G1557:2G |
| NZ_LS483377.1 | 3824004 | syn | smeE | A<->G | N1554:2N |
| NZ_LS483377.1 | 3824037 | syn | smeE | G<->A | V1521:2V |
| NZ_LS483377.1 | 3824040 | syn | smeE | G<->A | H1518:2H |
| NZ_LS483377.1 | 3824043 | syn | smeE | G<->A | H1515:2H |
| NZ_LS483377.1 | 3824072 | nonsyn | smeE | T<->C | M1486:0V |
| NZ_LS483377.1 | 3824091 | syn | smeE | G<->C | T1467:2T |
| NZ_LS483377.1 | 3824106 | syn | smeE | G<->C | V1452:2V |
| NZ_LS483377.1 | 3824121 | syn | smeE | T<->C | A1437:2A |
| NZ_LS483377.1 | 3824127 | syn | smeE | C<->T | A1431:2A |
| NZ_LS483377.1 | 3824130 | syn | smeE | C<->A | S1428:2S |
| NZ_LS483377.1 | 3824136 | syn | smeE | G<->A | I1422:2I |
| NZ_LS483377.1 | 3824154 | syn | smeE | G<->C | R1404:2R |
| NZ_LS483377.1 | 3824175 | syn | smeE | G<->T | G1383:2G |
| NZ_LS483377.1 | 3824178 | syn | smeE | A<->G | S1380:2S |
| NZ_LS483377.1 | 3824226 | syn | smeE | G<->A | G1332:2G |
| NZ_LS483377.1 | 3824238 | syn | smeE | G<->A | G1320:2G |
| NZ_LS483377.1 | 3824271 | syn | smeE | C<->T | E1287:2E |
| NZ_LS483377.1 | 3824274 | syn | smeE | G<->A | L1284:2L |
| NZ_LS483377.1 | 3824358 | syn | smeE | A<->C | L1200:2L |
| NZ_LS483377.1 | 3824400 | syn | smeE | G<->C | L1158:2L |
| NZ_LS483377.1 | 3824406 | syn | smeE | G<->C | A1152:2A |
| NZ_LS483377.1 | 3824525 | nonsyn | smeE | T<->G | I1033:0L |
| NZ_LS483377.1 | 3824585 | nonsyn | smeE | C<->T | V973:0I |
| NZ_LS483377.1 | 3824634 | syn | smeE | G<->A | A924:2A |
| NZ_LS483377.1 | 3824637 | syn | smeE | A<->G | N921:2N |
| NZ_LS483377.1 | 3824639 | nonsyn | smeE | T<->C | N919:0D |
| NZ_LS483377.1 | 3824643 | syn | smeE | A<->G | G915:2G |
| NZ_LS483377.1 | 3824652 | syn | smeE | G<->A | T906:2T |
| NZ_LS483377.1 | 3824685 | syn | smeE | C<->G | V873:2V |
| NZ_LS483377.1 | 3824751 | syn | smeE | C<->T | E807:2E |
| NZ_LS483377.1 | 3824769 | syn | smeE | C<->A | G789:2G |
| NZ_LS483377.1 | 3824799 | syn | smeE | A<->G | G759:2G |
| NZ_LS483377.1 | 3824883 | nonsyn | smeE | G<->C | I675:2M |
| NZ_LS483377.1 | 3824885 | nonsyn | smeE | T<->C | I673:0V |
| NZ_LS483377.1 | 3824892 | syn | smeE | T<->C | A666:2A |
| NZ_LS483377.1 | 3824901 | syn | smeE | A<->G | L657:2L |
| NZ_LS483377.1 | 3824913 | syn | smeE | G<->C | A645:2A |
| NZ_LS483377.1 | 3824922 | syn | smeE | C<->G | A636:2A |
| NZ_LS483377.1 | 3824925 | syn | smeE | G<->A | N633:2N |
| NZ_LS483377.1 | 3824940 | syn | smeE | C<->T | A618:2A |
| NZ_LS483377.1 | 3824943 | syn | smeE | T<->C | A615:2A |
| NZ_LS483377.1 | 3824946 | syn | smeE | G<->A | T612:2T |
| NZ_LS483377.1 | 3824958 | syn | smeE | A<->G | V600:2V |
| NZ_LS483377.1 | 3824961 | syn | smeE | C<->G | S597:2S |
| NZ_LS483377.1 | 3824991 | syn | smeE | A<->G | D567:2D |
| NZ_LS483377.1 | 3825108 | syn | smeE | A<->G | D450:2D |
| NZ_LS483377.1 | 3825192 | syn | smeE | A<->C | V366:2V |
| NZ_LS483377.1 | 3825255 | syn | smeE | G<->A | D303:2D |
| NZ_LS483377.1 | 3825384 | syn | smeE | C<->T | E174:2E |
| NZ_LS483377.1 | 3825435 | syn | smeE | C<->T | P123:2P |
| NZ_LS483377.1 | 3825441 | syn | smeE | T<->C | A117:2A |
| NZ_LS483377.1 | 3825492 | syn | smeE | G<->A | A66:2A |
| NZ_LS483377.1 | 3821018 | syn | smeF | G<->C | T1359:2T |
| NZ_LS483377.1 | 3821081 | syn | smeF | C<->G | T1296:2T |
| NZ_LS483377.1 | 3821087 | syn | smeF | C<->T | R1290:2R |
| NZ_LS483377.1 | 3821576 | syn | smeF | A<->C | L801:2L |
| NZ_LS483377.1 | 3821588 | syn | smeF | C<->T | E789:2E |
| NZ_LS483377.1 | 3821596 | nonsyn | smeF | T<->C | S781:0G |
| NZ_LS483377.1 | 3821609 | syn | smeF | C<->T | A768:2A |
| NZ_LS483377.1 | 3821618 | syn | smeF | C<->G | L759:2L |
| NZ_LS483377.1 | 3821681 | syn | smeF | T<->C | A696:2A |
| NZ_LS483377.1 | 3821684 | syn | smeF | G<->A | A693:2A |
| NZ_LS483377.1 | 3821687 | syn | smeF | G<->A | D690:2D |
| NZ_LS483377.1 | 3821690 | syn | smeF | C<->G | T687:2T |
| NZ_LS483377.1 | 3821693 | syn | smeF | A<->G | R684:2R |
| NZ_LS483377.1 | 3821711 | syn | smeF | G<->C | T666:2T |
| NZ_LS483377.1 | 3821714 | syn | smeF | A<->G | R663:2R |
| NZ_LS483377.1 | 3821729 | syn | smeF | C<->T | E648:2E |
| NZ_LS483377.1 | 3821735 | syn | smeF | C<->T | A642:2A |
| NZ_LS483377.1 | 3821747 | syn | smeF | A<->G | G630:2G |
| NZ_LS483377.1 | 3821753 | syn | smeF | T<->C | E624:2E |
| NZ_LS483377.1 | 3821779 | syn | smeF | G<->A | L598:0L |
| NZ_LS483377.1 | 3821786 | syn | smeF | C<->T | E591:2E |
| NZ_LS483377.1 | 3821807 | nonsyn | smeF | A<->C | D570:2E |
| NZ_LS483377.1 | 3821822 | syn | smeF | C<->G | R555:2R |
| NZ_LS483377.1 | 3821840 | syn | smeF | G<->A | Y537:2Y |
| NZ_LS483377.1 | 3821843 | syn | smeF | G<->C | T534:2T |
| NZ_LS483377.1 | 3821852 | syn | smeF | T<->C | A525:2A |
| NZ_LS483377.1 | 3821855 | syn | smeF | G<->A | T522:2T |
| NZ_LS483377.1 | 3821858 | syn | smeF | G<->T | A519:2A |
| NZ_LS483377.1 | 3821861 | syn | smeF | C<->G | T516:2T |
| NZ_LS483377.1 | 3821870 | syn | smeF | C<->A | V507:2V |
| NZ_LS483377.1 | 3821963 | syn | smeF | A<->G | D414:2D |
| NZ_LS483377.1 | 3821987 | syn | smeF | G<->A | V390:2V |
| NZ_LS483377.1 | 3821990 | syn | smeF | A<->G | G387:2G |
| NZ_LS483377.1 | 3822005 | syn | smeF | T<->C | E372:2E |
| NZ_LS483377.1 | 3822009 | nonsyn | smeF | T<->C | N368:1S |
| NZ_LS483377.1 | 3822020 | syn | smeF | G<->A | D357:2D |
| NZ_LS483377.1 | 3822026 | syn | smeF | G<->A | G351:2G |
| NZ_LS483377.1 | 3822029 | nonsyn | smeF | C<->G | Q348:2H |
| NZ_LS483377.1 | 3822030 | nonsyn | smeF | T<->C | Q347:1R |
| NZ_LS483377.1 | 3822032 | syn | smeF | G<->A | R345:2R |
| NZ_LS483377.1 | 3822035 | nonsyn | smeF | C<->G | Q342:2H |
| NZ_LS483377.1 | 3822037 | nonsyn | smeF | G<->C | Q340:0E |
| NZ_LS483377.1 | 3822050 | syn | smeF | A<->G | V327:2V |
| NZ_LS483377.1 | 3822062 | syn | smeF | G<->A | P315:2P |
| NZ_LS483377.1 | 3822086 | syn | smeF | A<->G | R291:2R |
| NZ_LS483377.1 | 3822107 | syn | smeF | C<->T | E270:2E |
| NZ_LS483377.1 | 3822134 | syn | smeF | A<->G | D243:2D |
| NZ_LS483377.1 | 3822140 | syn | smeF | G<->A | N237:2N |
| NZ_LS483377.1 | 3822158 | nonsyn | smeF | G<->C | D219:2E |
| NZ_LS483377.1 | 3822216 | nonsyn | smeF | A<->G | V161:1A |
| NZ_LS483377.1 | 3822308 | syn | smeF | G<->A | G69:2G |
| NZ_LS483377.1 | 3822311 | syn | smeF | G<->T | A66:2A |
| NZ_LS483377.1 | 3822314 | syn | smeF | G<->C | L63:2L |
| NZ_LS483377.1 | 3822324 | nonsyn | smeF | A<->G | V53:1A |
| NZ_LS483377.1 | 3822326 | syn | smeF | A<->G | A51:2A |
| NZ_LS483377.1 | 3821081 | syn | smeF | C<->G | T1296:2T |
| NZ_LS483377.1 | 3821087 | syn | smeF | C<->T | R1290:2R |
| NZ_LS483377.1 | 3821576 | syn | smeF | A<->C | L801:2L |
| NZ_LS483377.1 | 3821588 | syn | smeF | C<->T | E789:2E |
| NZ_LS483377.1 | 3821596 | nonsyn | smeF | T<->C | S781:0G |
| NZ_LS483377.1 | 3821609 | syn | smeF | C<->T | A768:2A |
| NZ_LS483377.1 | 3821618 | syn | smeF | C<->G | L759:2L |
| NZ_LS483377.1 | 3821681 | syn | smeF | T<->C | A696:2A |
| NZ_LS483377.1 | 3821684 | syn | smeF | G<->A | A693:2A |
| NZ_LS483377.1 | 3821687 | syn | smeF | G<->A | D690:2D |
| NZ_LS483377.1 | 3821690 | syn | smeF | C<->G | T687:2T |
| NZ_LS483377.1 | 3821693 | syn | smeF | A<->G | R684:2R |
| NZ_LS483377.1 | 3821711 | syn | smeF | G<->C | T666:2T |
| NZ_LS483377.1 | 3821714 | syn | smeF | A<->G | R663:2R |
| NZ_LS483377.1 | 3821729 | syn | smeF | C<->T | E648:2E |
| NZ_LS483377.1 | 3821735 | syn | smeF | C<->T | A642:2A |
| NZ_LS483377.1 | 3821747 | syn | smeF | A<->G | G630:2G |
| NZ_LS483377.1 | 3821753 | syn | smeF | T<->C | E624:2E |
| NZ_LS483377.1 | 3821779 | syn | smeF | G<->A | L598:0L |
| NZ_LS483377.1 | 3821786 | syn | smeF | C<->T | E591:2E |
| NZ_LS483377.1 | 3821807 | nonsyn | smeF | A<->C | D570:2E |
| NZ_LS483377.1 | 3821822 | syn | smeF | C<->G | R555:2R |
| NZ_LS483377.1 | 3821840 | syn | smeF | G<->A | Y537:2Y |
| NZ_LS483377.1 | 3821843 | syn | smeF | G<->C | T534:2T |
| NZ_LS483377.1 | 3821852 | syn | smeF | T<->C | A525:2A |
| NZ_LS483377.1 | 3821855 | syn | smeF | G<->A | T522:2T |
| NZ_LS483377.1 | 3821858 | syn | smeF | G<->T | A519:2A |
| NZ_LS483377.1 | 3821861 | syn | smeF | C<->G | T516:2T |
| NZ_LS483377.1 | 3821870 | syn | smeF | C<->A | V507:2V |
| NZ_LS483377.1 | 3821963 | syn | smeF | A<->G | D414:2D |
| NZ_LS483377.1 | 3821987 | syn | smeF | G<->A | V390:2V |
| NZ_LS483377.1 | 3821990 | syn | smeF | A<->G | G387:2G |
| NZ_LS483377.1 | 3822005 | syn | smeF | T<->C | E372:2E |
| NZ_LS483377.1 | 3822009 | nonsyn | smeF | T<->C | N368:1S |
| NZ_LS483377.1 | 3822020 | syn | smeF | G<->A | D357:2D |
| NZ_LS483377.1 | 3822026 | syn | smeF | G<->A | G351:2G |
| NZ_LS483377.1 | 3822029 | nonsyn | smeF | C<->G | Q348:2H |
| NZ_LS483377.1 | 3822030 | nonsyn | smeF | T<->C | Q347:1R |
| NZ_LS483377.1 | 3822032 | syn | smeF | G<->A | R345:2R |
| NZ_LS483377.1 | 3822035 | nonsyn | smeF | C<->G | Q342:2H |
| NZ_LS483377.1 | 3822037 | nonsyn | smeF | G<->C | Q340:0E |
| NZ_LS483377.1 | 3822050 | syn | smeF | A<->G | V327:2V |
| NZ_LS483377.1 | 3822062 | syn | smeF | G<->A | P315:2P |
| NZ_LS483377.1 | 3822086 | syn | smeF | A<->G | R291:2R |
| NZ_LS483377.1 | 3822107 | syn | smeF | C<->T | E270:2E |
| NZ_LS483377.1 | 3822134 | syn | smeF | A<->G | D243:2D |
| NZ_LS483377.1 | 3822140 | syn | smeF | G<->A | N237:2N |
| NZ_LS483377.1 | 3822158 | nonsyn | smeF | G<->C | D219:2E |
| NZ_LS483377.1 | 3822216 | nonsyn | smeF | A<->G | V161:1A |
| NZ_LS483377.1 | 3822308 | syn | smeF | G<->A | G69:2G |
| NZ_LS483377.1 | 3822311 | syn | smeF | G<->T | A66:2A |
| NZ_LS483377.1 | 3822314 | syn | smeF | G<->C | L63:2L |
| NZ_LS483377.1 | 3822324 | nonsyn | smeF | A<->G | V53:1A |
| NZ_LS483377.1 | 3822326 | syn | smeF | A<->G | A51:2A |
| NZ_LS483377.1 | 3822501 | syn | smeE | A<->G | G3057:2G |
| NZ_LS483377.1 | 3822573 | syn | smeE | A<->G | G2985:2G |
| NZ_LS483377.1 | 3822615 | syn | smeE | C<->G | L2943:2L |
| NZ_LS483377.1 | 3822636 | syn | smeE | A<->G | R2922:2R |
| NZ_LS483377.1 | 3822657 | syn | smeE | A<->G | H2901:2H |
| NZ_LS483377.1 | 3822669 | syn | smeE | T<->C | E2889:2E |
| NZ_LS483377.1 | 3822680 | nonsyn | smeE | C<->T | G2878:0S |
| NZ_LS483377.1 | 3822681 | syn | smeE | T<->G | A2877:2A |
| NZ_LS483377.1 | 3822690 | syn | smeE | C<->T | E2868:2E |
| NZ_LS483377.1 | 3822699 | syn | smeE | C<->T | E2859:2E |
| NZ_LS483377.1 | 3822714 | syn | smeE | C<->G | V2844:2V |
| NZ_LS483377.1 | 3822774 | syn | smeE | G<->A | Y2784:2Y |
| NZ_LS483377.1 | 3822780 | syn | smeE | A<->G | D2778:2D |
| NZ_LS483377.1 | 3822786 | syn | smeE | T<->C | E2772:2E |
| NZ_LS483377.1 | 3822804 | syn | smeE | A<->G | N2754:2N |
| NZ_LS483377.1 | 3822828 | syn | smeE | G<->A | G2730:2G |
| NZ_LS483377.1 | 3822852 | syn | smeE | T<->C | A2706:2A |
| NZ_LS483377.1 | 3822861 | syn | smeE | C<->T | V2697:2V |
| NZ_LS483377.1 | 3822876 | syn | smeE | G<->A | Y2682:2Y |
| NZ_LS483377.1 | 3822900 | syn | smeE | T<->C | V2658:2V |
| NZ_LS483377.1 | 3822915 | syn | smeE | G<->C | L2643:2L |
| NZ_LS483377.1 | 3822945 | syn | smeE | A<->G | A2613:2A |
| NZ_LS483377.1 | 3822954 | syn | smeE | C<->T | E2604:2E |
| NZ_LS483377.1 | 3822978 | syn | smeE | T<->C | E2580:2E |
| NZ_LS483377.1 | 3822999 | syn | smeE | C<->A | L2559:2L |
| NZ_LS483377.1 | 3823017 | syn | smeE | T<->C | E2541:2E |
| NZ_LS483377.1 | 3823038 | syn | smeE | A<->G | G2520:2G |
| NZ_LS483377.1 | 3823074 | syn | smeE | T<->C | E2484:2E |
| NZ_LS483377.1 | 3823080 | syn | smeE | G<->C | A2478:2A |
| NZ_LS483377.1 | 3823101 | syn | smeE | T<->C | E2457:2E |
| NZ_LS483377.1 | 3823143 | syn | smeE | G<->T | A2415:2A |
| NZ_LS483377.1 | 3823161 | syn | smeE | T<->C | E2397:2E |
| NZ_LS483377.1 | 3823164 | syn | smeE | A<->G | G2394:2G |
| NZ_LS483377.1 | 3823191 | syn | smeE | A<->G | D2367:2D |
| NZ_LS483377.1 | 3823206 | syn | smeE | C<->G | V2352:2V |
| NZ_LS483377.1 | 3823242 | syn | smeE | A<->G | R2316:2R |
| NZ_LS483377.1 | 3823272 | syn | smeE | G<->A | D2286:2D |
| NZ_LS483377.1 | 3823296 | syn | smeE | G<->A | A2262:2A |
| NZ_LS483377.1 | 3823434 | syn | smeE | G<->C | L2124:2L |
| NZ_LS483377.1 | 3823455 | syn | smeE | C<->T | A2103:2A |
| NZ_LS483377.1 | 3823479 | syn | smeE | C<->T | Q2079:2Q |
| NZ_LS483377.1 | 3823524 | syn | smeE | G<->A | G2034:2G |
| NZ_LS483377.1 | 3823536 | syn | smeE | G<->A | I2022:2I |
| NZ_LS483377.1 | 3823557 | syn | smeE | G<->A | F2001:2F |
| NZ_LS483377.1 | 3823599 | syn | smeE | G<->A | R1959:2R |
| NZ_LS483377.1 | 3823602 | syn | smeE | T<->A | G1956:2G |
| NZ_LS483377.1 | 3823628 | nonsyn | smeE | T<->C | N1930:0D |
| NZ_LS483377.1 | 3823713 | syn | smeE | C<->G | S1845:2S |
| NZ_LS483377.1 | 3823725 | syn | smeE | G<->A | D1833:2D |
| NZ_LS483377.1 | 3823761 | syn | smeE | T<->C | E1797:2E |
| NZ_LS483377.1 | 3823766 | syn | smeE | G<->A | L1792:0L |
| NZ_LS483377.1 | 3823788 | syn | smeE | A<->G | R1770:2R |
| NZ_LS483377.1 | 3823791 | syn | smeE | T<->C | E1767:2E |
| NZ_LS483377.1 | 3823803 | syn | smeE | A<->G | G1755:2G |
| NZ_LS483377.1 | 3823806 | syn | smeE | C<->G | V1752:2V |
| NZ_LS483377.1 | 3823832 | syn | smeE | A<->G | L1726:0L |
| NZ_LS483377.1 | 3823860 | syn | smeE | T<->C | S1698:2S |
| NZ_LS483377.1 | 3823883 | syn | smeE | A<->G | L1675:0L |
| NZ_LS483377.1 | 3823905 | syn | smeE | C<->T | A1653:2A |
| NZ_LS483377.1 | 3823935 | syn | smeE | G<->A | R1623:2R |
| NZ_LS483377.1 | 3823956 | syn | smeE | G<->A | G1602:2G |
| NZ_LS483377.1 | 3823968 | syn | smeE | G<->A | S1590:2S |
| NZ_LS483377.1 | 3823989 | syn | smeE | T<->G | G1569:2G |
| NZ_LS483377.1 | 3824001 | syn | smeE | G<->A | G1557:2G |
| NZ_LS483377.1 | 3824004 | syn | smeE | A<->G | N1554:2N |
| NZ_LS483377.1 | 3824037 | syn | smeE | G<->A | V1521:2V |
| NZ_LS483377.1 | 3824040 | syn | smeE | G<->A | H1518:2H |
| NZ_LS483377.1 | 3824043 | syn | smeE | G<->A | H1515:2H |
| NZ_LS483377.1 | 3824072 | nonsyn | smeE | T<->C | M1486:0V |
| NZ_LS483377.1 | 3824091 | syn | smeE | G<->C | T1467:2T |
| NZ_LS483377.1 | 3824106 | syn | smeE | G<->C | V1452:2V |
| NZ_LS483377.1 | 3824121 | syn | smeE | T<->C | A1437:2A |
| NZ_LS483377.1 | 3824127 | syn | smeE | C<->T | A1431:2A |
| NZ_LS483377.1 | 3824130 | syn | smeE | C<->A | S1428:2S |
| NZ_LS483377.1 | 3824136 | syn | smeE | G<->A | I1422:2I |
| NZ_LS483377.1 | 3824154 | syn | smeE | G<->C | R1404:2R |
| NZ_LS483377.1 | 3824175 | syn | smeE | G<->T | G1383:2G |
| NZ_LS483377.1 | 3824178 | syn | smeE | A<->G | S1380:2S |
| NZ_LS483377.1 | 3824226 | syn | smeE | G<->A | G1332:2G |
| NZ_LS483377.1 | 3824238 | syn | smeE | G<->A | G1320:2G |
| NZ_LS483377.1 | 3824271 | syn | smeE | C<->T | E1287:2E |
| NZ_LS483377.1 | 3824274 | syn | smeE | G<->A | L1284:2L |
| NZ_LS483377.1 | 3824358 | syn | smeE | A<->C | L1200:2L |
| NZ_LS483377.1 | 3824400 | syn | smeE | G<->C | L1158:2L |
| NZ_LS483377.1 | 3824406 | syn | smeE | G<->C | A1152:2A |
| NZ_LS483377.1 | 3824525 | nonsyn | smeE | T<->G | I1033:0L |
| NZ_LS483377.1 | 3824585 | nonsyn | smeE | C<->T | V973:0I |
| NZ_LS483377.1 | 3824634 | syn | smeE | G<->A | A924:2A |
| NZ_LS483377.1 | 3824637 | syn | smeE | A<->G | N921:2N |
| NZ_LS483377.1 | 3824639 | nonsyn | smeE | T<->C | N919:0D |
| NZ_LS483377.1 | 3824643 | syn | smeE | A<->G | G915:2G |
| NZ_LS483377.1 | 3824652 | syn | smeE | G<->A | T906:2T |
| NZ_LS483377.1 | 3824685 | syn | smeE | C<->G | V873:2V |
| NZ_LS483377.1 | 3824751 | syn | smeE | C<->T | E807:2E |
| NZ_LS483377.1 | 3824769 | syn | smeE | C<->A | G789:2G |
| NZ_LS483377.1 | 3824799 | syn | smeE | A<->G | G759:2G |
| NZ_LS483377.1 | 3824883 | nonsyn | smeE | G<->C | I675:2M |
| NZ_LS483377.1 | 3824885 | nonsyn | smeE | T<->C | I673:0V |
| NZ_LS483377.1 | 3824892 | syn | smeE | T<->C | A666:2A |
| NZ_LS483377.1 | 3824901 | syn | smeE | A<->G | L657:2L |
| NZ_LS483377.1 | 3824913 | syn | smeE | G<->C | A645:2A |
| NZ_LS483377.1 | 3824922 | syn | smeE | C<->G | A636:2A |
| NZ_LS483377.1 | 3824925 | syn | smeE | G<->A | N633:2N |
| NZ_LS483377.1 | 3824940 | syn | smeE | C<->T | A618:2A |
| NZ_LS483377.1 | 3824943 | syn | smeE | T<->C | A615:2A |
| NZ_LS483377.1 | 3824946 | syn | smeE | G<->A | T612:2T |
| NZ_LS483377.1 | 3824958 | syn | smeE | A<->G | V600:2V |
| NZ_LS483377.1 | 3824961 | syn | smeE | C<->G | S597:2S |
| NZ_LS483377.1 | 3824991 | syn | smeE | A<->G | D567:2D |
| NZ_LS483377.1 | 3825108 | syn | smeE | A<->G | D450:2D |
| NZ_LS483377.1 | 3825192 | syn | smeE | A<->C | V366:2V |
| NZ_LS483377.1 | 3825255 | syn | smeE | G<->A | D303:2D |
| NZ_LS483377.1 | 3825384 | syn | smeE | C<->T | E174:2E |
| NZ_LS483377.1 | 3825435 | syn | smeE | C<->T | P123:2P |
| NZ_LS483377.1 | 3825441 | syn | smeE | T<->C | A117:2A |
| NZ_LS483377.1 | 3825492 | syn | smeE | G<->A | A66:2A |
| NZ_LS483377.1 | 3825606 | syn | smeD | G<->A | A1149:2A |
| NZ_LS483377.1 | 3825607 | nonsyn | smeD | G<->T | A1148:1D |
| NZ_LS483377.1 | 3825609 | syn | smeD | C<->G | A1146:2A |
| NZ_LS483377.1 | 3825612 | syn | smeD | T<->C | A1143:2A |
| NZ_LS483377.1 | 3825627 | syn | smeD | A<->T | A1128:2A |
| NZ_LS483377.1 | 3825630 | syn | smeD | A<->G | A1125:2A |
| NZ_LS483377.1 | 3825631 | nonsyn | smeD | G<->T | A1124:1D |
| NZ_LS483377.1 | 3825639 | syn | smeD | C<->T | E1116:2E |
| NZ_LS483377.1 | 3825687 | syn | smeD | G<->A | V1068:2V |
| NZ_LS483377.1 | 3825702 | syn | smeD | G<->A | G1053:2G |
| NZ_LS483377.1 | 3825714 | syn | smeD | A<->G | G1041:2G |
| NZ_LS483377.1 | 3825846 | syn | smeD | T<->C | V909:2V |
| NZ_LS483377.1 | 3825888 | syn | smeD | G<->A | R867:2R |
| NZ_LS483377.1 | 3825897 | syn | smeD | G<->C | T858:2T |
| NZ_LS483377.1 | 3825898 | nonsyn | smeD | G<->A | T857:1I |
| NZ_LS483377.1 | 3825944 | syn | smeD | G<->A | L811:0L |
| NZ_LS483377.1 | 3825962 | nonsyn | smeD | C<->T | A793:0T |
| NZ_LS483377.1 | 3825963 | syn | smeD | T<->C | P792:2P |
| NZ_LS483377.1 | 3825975 | syn | smeD | C<->G | V780:2V |
| NZ_LS483377.1 | 3825981 | syn | smeD | A<->G | S774:2S |
| NZ_LS483377.1 | 3826047 | syn | smeD | C<->G | T708:2T |
| NZ_LS483377.1 | 3826101 | syn | smeD | C<->T | E654:2E |
| NZ_LS483377.1 | 3826107 | syn | smeD | C<->G | S648:2S |
| NZ_LS483377.1 | 3826128 | syn | smeD | G<->A | Y627:2Y |
| NZ_LS483377.1 | 3826155 | syn | smeD | G<->A | A600:2A |
| NZ_LS483377.1 | 3826200 | syn | smeD | A<->G | V555:2V |
| NZ_LS483377.1 | 3826326 | syn | smeD | C<->G | V429:2V |
| NZ_LS483377.1 | 3826488 | syn | smeD | G<->C | L267:2L |
| NZ_LS483377.1 | 3826614 | syn | smeD | G<->A | G141:2G |
| NZ_LS483377.1 | 3826623 | syn | smeD | C<->T | E132:2E |
| NZ_LS483377.1 | 3826680 | syn | smeD | T<->C | Q75:2Q |

Table S2 SNPs that WJ_18a has compared to WJ_18

| Chromosomes | Position | Type of SNP | Gene | Mutation mode | Amino acid mutations |
| --- | --- | --- | --- | --- | --- |
| NZ_LS483377.1 | 3826165 | nonsyn | smeD | T<->C | N590:1S |
| NZ_LS483377.1 | 3822702 | syn | smeE | C<->T | K2856:2K |
| NZ_LS483377.1 | 3822963 | syn | smeE | C<->T | S2595:2S |
| NZ_LS483377.1 | 3823137 | syn | smeE | A<->G | A2421:2A |
| NZ_LS483377.1 | 3823326 | syn | smeE | T<->C | S2232:2S |
| NZ_LS483377.1 | 3823467 | syn | smeE | T<->C | A2091:2A |
| NZ_LS483377.1 | 3823771 | nonsyn | smeE | G<->T | A1787:1E |
| NZ_LS483377.1 | 3823812 | syn | smeE | G<->C | A1746:2A |
| NZ_LS483377.1 | 3824052 | syn | smeE | C<->T | K1506:2K |
| NZ_LS483377.1 | 3824646 | syn | smeE | A<->G | A912:2A |
| NZ_LS483377.1 | 3824649 | syn | smeE | A<->G | A909:2A |
| NZ_LS483377.1 | 3821005 | nonsyn | smeF | T<->G | M1372:0L |
| NZ_LS483377.1 | 3821066 | syn | smeF | T<->C | Q1311:2Q |
| NZ_LS483377.1 | 3821246 | syn | smeF | G<->A | G1131:2G |
| NZ_LS483377.1 | 3821294 | syn | smeF | C<->G | A1083:2A |
| NZ_LS483377.1 | 3821510 | syn | smeF | G<->A | A867:2A |
| NZ_LS483377.1 | 3821906 | syn | smeF | C<->G | V471:2V |
| NZ_LS483377.1 | 3821909 | syn | smeF | C<->G | A468:2A |
| NZ_LS483377.1 | 3821930 | syn | smeF | A<->G | A447:2A |
| NZ_LS483377.1 | 3822242 | syn | smeF | C<->T | E135:2E |
| NZ_LS483377.1 | 3821246 | syn | smeF | G<->A | G1131:2G |
| NZ_LS483377.1 | 3821294 | syn | smeF | C<->G | A1083:2A |
| NZ_LS483377.1 | 3821510 | syn | smeF | G<->A | A867:2A |
| NZ_LS483377.1 | 3821906 | syn | smeF | C<->G | V471:2V |
| NZ_LS483377.1 | 3821909 | syn | smeF | C<->G | A468:2A |
| NZ_LS483377.1 | 3821930 | syn | smeF | A<->G | A447:2A |
| NZ_LS483377.1 | 3822242 | syn | smeF | C<->T | E135:2E |
| NZ_LS483377.1 | 3822702 | syn | smeE | C<->T | K2856:2K |
| NZ_LS483377.1 | 3822963 | syn | smeE | C<->T | S2595:2S |
| NZ_LS483377.1 | 3823137 | syn | smeE | A<->G | A2421:2A |
| NZ_LS483377.1 | 3823326 | syn | smeE | T<->C | S2232:2S |
| NZ_LS483377.1 | 3823467 | syn | smeE | T<->C | A2091:2A |
| NZ_LS483377.1 | 3823771 | nonsyn | smeE | G<->T | A1787:1E |
| NZ_LS483377.1 | 3823812 | syn | smeE | G<->C | A1746:2A |
| NZ_LS483377.1 | 3824052 | syn | smeE | C<->T | K1506:2K |
| NZ_LS483377.1 | 3824646 | syn | smeE | A<->G | A912:2A |
| NZ_LS483377.1 | 3824649 | syn | smeE | A<->G | A909:2A |
| NZ_LS483377.1 | 3826165 | nonsyn | smeD | T<->C | N590:1S |

Table S3 SNPs with up-regulated expression in WJ_97 compared to the reference genome NCTC10258

| Chromosomes | Position | Type of SNP | Gene | Mutation mode | Amino acid mutations |
| --- | --- | --- | --- | --- | --- |
| NZ_LS483377.1 | 3822365 | Deletion | smeF | A | - |
| NZ_LS483377.1 | 3822365 | Deletion | smeF | A | - |
| NZ_LS483377.1 | 3822365 | Deletion | smeF | A | - |
| NZ_LS483377.1 | 3822365 | Deletion | smeF | A | - |
| NZ_LS483377.1 | 3820997 | syn | smeF | A<->G | G1380:2G |
| NZ_LS483377.1 | 3821005 | nonsyn | smeF | T<->G | M1372:0L |
| NZ_LS483377.1 | 3821017 | syn | smeF | G<->A | L1360:0L |
| NZ_LS483377.1 | 3821018 | syn | smeF | G<->C | T1359:2T |
| NZ_LS483377.1 | 3821039 | syn | smeF | C<->G | A1338:2A |
| NZ_LS483377.1 | 3821060 | syn | smeF | G<->C | T1317:2T |
| NZ_LS483377.1 | 3821066 | syn | smeF | T<->C | Q1311:2Q |
| NZ_LS483377.1 | 3821069 | syn | smeF | T<->C | A1308:2A |
| NZ_LS483377.1 | 3821069 | syn | smeF | T<->C | A1308:2A |
| NZ_LS483377.1 | 3821081 | syn | smeF | C<->G | T1296:2T |
| NZ_LS483377.1 | 3821081 | syn | smeF | C<->G | T1296:2T |
| NZ_LS483377.1 | 3821090 | syn | smeF | C<->T | A1287:2A |
| NZ_LS483377.1 | 3821090 | syn | smeF | C<->T | A1287:2A |
| NZ_LS483377.1 | 3821096 | syn | smeF | C<->G | L1281:2L |
| NZ_LS483377.1 | 3821096 | syn | smeF | C<->G | L1281:2L |
| NZ_LS483377.1 | 3821105 | syn | smeF | C<->G | V1272:2V |
| NZ_LS483377.1 | 3821105 | syn | smeF | C<->G | V1272:2V |
| NZ_LS483377.1 | 3821123 | syn | smeF | C<->G | A1254:2A |
| NZ_LS483377.1 | 3821123 | syn | smeF | C<->G | A1254:2A |
| NZ_LS483377.1 | 3821141 | syn | smeF | C<->T | S1236:2S |
| NZ_LS483377.1 | 3821141 | syn | smeF | C<->T | S1236:2S |
| NZ_LS483377.1 | 3821168 | nonsyn | smeF | T<->A | E1209:2D |
| NZ_LS483377.1 | 3821168 | nonsyn | smeF | T<->A | E1209:2D |
| NZ_LS483377.1 | 3821188 | nonsyn | smeF | A<->C | S1189:0A |
| NZ_LS483377.1 | 3821188 | nonsyn | smeF | A<->C | S1189:0A |
| NZ_LS483377.1 | 3821201 | syn | smeF | G<->A | D1176:2D |
| NZ_LS483377.1 | 3821201 | syn | smeF | G<->A | D1176:2D |
| NZ_LS483377.1 | 3821218 | syn | smeF | A<->G | L1159:0L |
| NZ_LS483377.1 | 3821218 | syn | smeF | A<->G | L1159:0L |
| NZ_LS483377.1 | 3821228 | syn | smeF | A<->G | D1149:2D |
| NZ_LS483377.1 | 3821228 | syn | smeF | A<->G | D1149:2D |
| NZ_LS483377.1 | 3821249 | syn | smeF | C<->G | V1128:2V |
| NZ_LS483377.1 | 3821249 | syn | smeF | C<->G | V1128:2V |
| NZ_LS483377.1 | 3821258 | syn | smeF | C<->T | S1119:2S |
| NZ_LS483377.1 | 3821258 | syn | smeF | C<->T | S1119:2S |
| NZ_LS483377.1 | 3821279 | syn | smeF | A<->C | A1098:2A |
| NZ_LS483377.1 | 3821279 | syn | smeF | A<->C | A1098:2A |
| NZ_LS483377.1 | 3821288 | syn | smeF | A<->G | R1089:2R |
| NZ_LS483377.1 | 3821288 | syn | smeF | A<->G | R1089:2R |
| NZ_LS483377.1 | 3821291 | syn | smeF | A<->G | D1086:2D |
| NZ_LS483377.1 | 3821291 | syn | smeF | A<->G | D1086:2D |
| NZ_LS483377.1 | 3821297 | syn | smeF | G<->A | N1080:2N |
| NZ_LS483377.1 | 3821297 | syn | smeF | G<->A | N1080:2N |
| NZ_LS483377.1 | 3821348 | syn | smeF | C<->G | T1029:2T |
| NZ_LS483377.1 | 3821348 | syn | smeF | C<->G | T1029:2T |
| NZ_LS483377.1 | 3821378 | syn | smeF | C<->G | T999:2T |
| NZ_LS483377.1 | 3821378 | syn | smeF | C<->G | T999:2T |
| NZ_LS483377.1 | 3821381 | syn | smeF | A<->G | G996:2G |
| NZ_LS483377.1 | 3821381 | syn | smeF | A<->G | G996:2G |
| NZ_LS483377.1 | 3821462 | syn | smeF | G<->A | R915:2R |
| NZ_LS483377.1 | 3821462 | syn | smeF | G<->A | R915:2R |
| NZ_LS483377.1 | 3821471 | syn | smeF | A<->G | G906:2G |
| NZ_LS483377.1 | 3821471 | syn | smeF | A<->G | G906:2G |
| NZ_LS483377.1 | 3821477 | syn | smeF | A<->G | N900:2N |
| NZ_LS483377.1 | 3821477 | syn | smeF | A<->G | N900:2N |
| NZ_LS483377.1 | 3821489 | syn | smeF | C<->A | A888:2A |
| NZ_LS483377.1 | 3821489 | syn | smeF | C<->A | A888:2A |
| NZ_LS483377.1 | 3821555 | syn | smeF | G<->A | A822:2A |
| NZ_LS483377.1 | 3821555 | syn | smeF | G<->A | A822:2A |
| NZ_LS483377.1 | 3821573 | syn | smeF | T<->C | A804:2A |
| NZ_LS483377.1 | 3821573 | syn | smeF | T<->C | A804:2A |
| NZ_LS483377.1 | 3821576 | syn | smeF | A<->C | L801:2L |
| NZ_LS483377.1 | 3821576 | syn | smeF | A<->C | L801:2L |
| NZ_LS483377.1 | 3821588 | syn | smeF | C<->T | E789:2E |
| NZ_LS483377.1 | 3821588 | syn | smeF | C<->T | E789:2E |
| NZ_LS483377.1 | 3821611 | nonsyn | smeF | C<->A | A766:0S |
| NZ_LS483377.1 | 3821611 | nonsyn | smeF | C<->A | A766:0S |
| NZ_LS483377.1 | 3821624 | syn | smeF | G<->A | G753:2G |
| NZ_LS483377.1 | 3821624 | syn | smeF | G<->A | G753:2G |
| NZ_LS483377.1 | 3821639 | syn | smeF | C<->T | A738:2A |
| NZ_LS483377.1 | 3821639 | syn | smeF | C<->T | A738:2A |
| NZ_LS483377.1 | 3821669 | syn | smeF | G<->C | G708:2G |
| NZ_LS483377.1 | 3821669 | syn | smeF | G<->C | G708:2G |
| NZ_LS483377.1 | 3821687 | syn | smeF | G<->A | D690:2D |
| NZ_LS483377.1 | 3821687 | syn | smeF | G<->A | D690:2D |
| NZ_LS483377.1 | 3821690 | syn | smeF | C<->G | T687:2T |
| NZ_LS483377.1 | 3821690 | syn | smeF | C<->G | T687:2T |
| NZ_LS483377.1 | 3821693 | syn | smeF | A<->G | R684:2R |
| NZ_LS483377.1 | 3821693 | syn | smeF | A<->G | R684:2R |
| NZ_LS483377.1 | 3821702 | syn | smeF | C<->T | E675:2E |
| NZ_LS483377.1 | 3821702 | syn | smeF | C<->T | E675:2E |
| NZ_LS483377.1 | 3821705 | syn | smeF | G<->C | V672:2V |
| NZ_LS483377.1 | 3821705 | syn | smeF | G<->C | V672:2V |
| NZ_LS483377.1 | 3821711 | syn | smeF | G<->C | T666:2T |
| NZ_LS483377.1 | 3821711 | syn | smeF | G<->C | T666:2T |
| NZ_LS483377.1 | 3821714 | syn | smeF | A<->G | R663:2R |
| NZ_LS483377.1 | 3821714 | syn | smeF | A<->G | R663:2R |
| NZ_LS483377.1 | 3821729 | syn | smeF | C<->T | E648:2E |
| NZ_LS483377.1 | 3821729 | syn | smeF | C<->T | E648:2E |
| NZ_LS483377.1 | 3821735 | syn | smeF | C<->T | A642:2A |
| NZ_LS483377.1 | 3821735 | syn | smeF | C<->T | A642:2A |
| NZ_LS483377.1 | 3821747 | syn | smeF | A<->G | G630:2G |
| NZ_LS483377.1 | 3821747 | syn | smeF | A<->G | G630:2G |
| NZ_LS483377.1 | 3821750 | syn | smeF | A<->G | R627:2R |
| NZ_LS483377.1 | 3821750 | syn | smeF | A<->G | R627:2R |
| NZ_LS483377.1 | 3821753 | syn | smeF | T<->C | E624:2E |
| NZ_LS483377.1 | 3821753 | syn | smeF | T<->C | E624:2E |
| NZ_LS483377.1 | 3821756 | syn | smeF | G<->A | H621:2H |
| NZ_LS483377.1 | 3821756 | syn | smeF | G<->A | H621:2H |
| NZ_LS483377.1 | 3821765 | syn | smeF | C<->T | E612:2E |
| NZ_LS483377.1 | 3821765 | syn | smeF | C<->T | E612:2E |
| NZ_LS483377.1 | 3821828 | syn | smeF | C<->T | A549:2A |
| NZ_LS483377.1 | 3821828 | syn | smeF | C<->T | A549:2A |
| NZ_LS483377.1 | 3821840 | syn | smeF | G<->A | Y537:2Y |
| NZ_LS483377.1 | 3821840 | syn | smeF | G<->A | Y537:2Y |
| NZ_LS483377.1 | 3821852 | syn | smeF | T<->C | A525:2A |
| NZ_LS483377.1 | 3821852 | syn | smeF | T<->C | A525:2A |
| NZ_LS483377.1 | 3821855 | syn | smeF | G<->C | T522:2T |
| NZ_LS483377.1 | 3821855 | syn | smeF | G<->C | T522:2T |
| NZ_LS483377.1 | 3821861 | syn | smeF | C<->G | T516:2T |
| NZ_LS483377.1 | 3821861 | syn | smeF | C<->G | T516:2T |
| NZ_LS483377.1 | 3821881 | syn | smeF | A<->G | L496:0L |
| NZ_LS483377.1 | 3821881 | syn | smeF | A<->G | L496:0L |
| NZ_LS483377.1 | 3821888 | syn | smeF | G<->A | N489:2N |
| NZ_LS483377.1 | 3821888 | syn | smeF | G<->A | N489:2N |
| NZ_LS483377.1 | 3821897 | syn | smeF | G<->A | N480:2N |
| NZ_LS483377.1 | 3821897 | syn | smeF | G<->A | N480:2N |
| NZ_LS483377.1 | 3821903 | syn | smeF | G<->C | A474:2A |
| NZ_LS483377.1 | 3821903 | syn | smeF | G<->C | A474:2A |
| NZ_LS483377.1 | 3821909 | syn | smeF | C<->A | A468:2A |
| NZ_LS483377.1 | 3821909 | syn | smeF | C<->A | A468:2A |
| NZ_LS483377.1 | 3821924 | syn | smeF | T<->C | L453:2L |
| NZ_LS483377.1 | 3821924 | syn | smeF | T<->C | L453:2L |
| NZ_LS483377.1 | 3821930 | syn | smeF | A<->T | A447:2A |
| NZ_LS483377.1 | 3821930 | syn | smeF | A<->T | A447:2A |
| NZ_LS483377.1 | 3821933 | syn | smeF | T<->C | E444:2E |
| NZ_LS483377.1 | 3821933 | syn | smeF | T<->C | E444:2E |
| NZ_LS483377.1 | 3821936 | syn | smeF | A<->G | S441:2S |
| NZ_LS483377.1 | 3821936 | syn | smeF | A<->G | S441:2S |
| NZ_LS483377.1 | 3821942 | syn | smeF | A<->G | N435:2N |
| NZ_LS483377.1 | 3821942 | syn | smeF | A<->G | N435:2N |
| NZ_LS483377.1 | 3821963 | syn | smeF | A<->G | D414:2D |
| NZ_LS483377.1 | 3821963 | syn | smeF | A<->G | D414:2D |
| NZ_LS483377.1 | 3821984 | syn | smeF | G<->A | G393:2G |
| NZ_LS483377.1 | 3821984 | syn | smeF | G<->A | G393:2G |
| NZ_LS483377.1 | 3821987 | syn | smeF | G<->C | V390:2V |
| NZ_LS483377.1 | 3821987 | syn | smeF | G<->C | V390:2V |
| NZ_LS483377.1 | 3821990 | syn | smeF | A<->G | G387:2G |
| NZ_LS483377.1 | 3821990 | syn | smeF | A<->G | G387:2G |
| NZ_LS483377.1 | 3821993 | syn | smeF | G<->C | A384:2A |
| NZ_LS483377.1 | 3821993 | syn | smeF | G<->C | A384:2A |
| NZ_LS483377.1 | 3822005 | syn | smeF | T<->C | E372:2E |
| NZ_LS483377.1 | 3822005 | syn | smeF | T<->C | E372:2E |
| NZ_LS483377.1 | 3822009 | nonsyn | smeF | T<->G | N368:1T |
| NZ_LS483377.1 | 3822009 | nonsyn | smeF | T<->G | N368:1T |
| NZ_LS483377.1 | 3822014 | syn | smeF | A<->G | G363:2G |
| NZ_LS483377.1 | 3822014 | syn | smeF | A<->G | G363:2G |
| NZ_LS483377.1 | 3822037 | nonsyn | smeF | G<->C | Q340:0E |
| NZ_LS483377.1 | 3822037 | nonsyn | smeF | G<->C | Q340:0E |
| NZ_LS483377.1 | 3822050 | syn | smeF | A<->G | V327:2V |
| NZ_LS483377.1 | 3822050 | syn | smeF | A<->G | V327:2V |
| NZ_LS483377.1 | 3822065 | syn | smeF | C<->T | V312:2V |
| NZ_LS483377.1 | 3822065 | syn | smeF | C<->T | V312:2V |
| NZ_LS483377.1 | 3822071 | syn | smeF | A<->G | D306:2D |
| NZ_LS483377.1 | 3822071 | syn | smeF | A<->G | D306:2D |
| NZ_LS483377.1 | 3822074 | syn | smeF | A<->G | A303:2A |
| NZ_LS483377.1 | 3822074 | syn | smeF | A<->G | A303:2A |
| NZ_LS483377.1 | 3822086 | syn | smeF | A<->G | R291:2R |
| NZ_LS483377.1 | 3822086 | syn | smeF | A<->G | R291:2R |
| NZ_LS483377.1 | 3822101 | syn | smeF | C<->T | A276:2A |
| NZ_LS483377.1 | 3822101 | syn | smeF | C<->T | A276:2A |
| NZ_LS483377.1 | 3822107 | syn | smeF | C<->T | E270:2E |
| NZ_LS483377.1 | 3822107 | syn | smeF | C<->T | E270:2E |
| NZ_LS483377.1 | 3822110 | syn | smeF | A<->C | V267:2V |
| NZ_LS483377.1 | 3822110 | syn | smeF | A<->C | V267:2V |
| NZ_LS483377.1 | 3822134 | syn | smeF | A<->G | D243:2D |
| NZ_LS483377.1 | 3822134 | syn | smeF | A<->G | D243:2D |
| NZ_LS483377.1 | 3822137 | syn | smeF | T<->G | R240:2R |
| NZ_LS483377.1 | 3822137 | syn | smeF | T<->G | R240:2R |
| NZ_LS483377.1 | 3822154 | nonsyn | smeF | T<->A | T223:0S |
| NZ_LS483377.1 | 3822154 | nonsyn | smeF | T<->A | T223:0S |
| NZ_LS483377.1 | 3822176 | syn | smeF | G<->T | R201:2R |
| NZ_LS483377.1 | 3822176 | syn | smeF | G<->T | R201:2R |
| NZ_LS483377.1 | 3822182 | syn | smeF | A<->G | D195:2D |
| NZ_LS483377.1 | 3822182 | syn | smeF | A<->G | D195:2D |
| NZ_LS483377.1 | 3822215 | syn | smeF | G<->A | V162:2V |
| NZ_LS483377.1 | 3822215 | syn | smeF | G<->A | V162:2V |
| NZ_LS483377.1 | 3822229 | nonsyn | smeF | C<->G | E148:0Q |
| NZ_LS483377.1 | 3822229 | nonsyn | smeF | C<->G | E148:0Q |
| NZ_LS483377.1 | 3822238 | nonsyn | smeF | C<->T | A139:0T |
| NZ_LS483377.1 | 3822238 | nonsyn | smeF | C<->T | A139:0T |
| NZ_LS483377.1 | 3822263 | syn | smeF | G<->A | I114:2I |
| NZ_LS483377.1 | 3822263 | syn | smeF | G<->A | I114:2I |
| NZ_LS483377.1 | 3822266 | syn | smeF | C<->G | A111:2A |
| NZ_LS483377.1 | 3822266 | syn | smeF | C<->G | A111:2A |
| NZ_LS483377.1 | 3822272 | syn | smeF | G<->C | A105:2A |
| NZ_LS483377.1 | 3822272 | syn | smeF | G<->C | A105:2A |
| NZ_LS483377.1 | 3822278 | syn | smeF | A<->G | A99:2A |
| NZ_LS483377.1 | 3822278 | syn | smeF | A<->G | A99:2A |
| NZ_LS483377.1 | 3822314 | syn | smeF | G<->C | L63:2L |
| NZ_LS483377.1 | 3822314 | syn | smeF | G<->C | L63:2L |
| NZ_LS483377.1 | 3822320 | syn | smeF | G<->A | L57:2L |
| NZ_LS483377.1 | 3822320 | syn | smeF | G<->A | L57:2L |
| NZ_LS483377.1 | 3822324 | nonsyn | smeF | A<->G | V53:1A |
| NZ_LS483377.1 | 3822324 | nonsyn | smeF | A<->G | V53:1A |
| NZ_LS483377.1 | 3822326 | syn | smeF | A<->G | A51:2A |
| NZ_LS483377.1 | 3822326 | syn | smeF | A<->G | A51:2A |
| NZ_LS483377.1 | 3822369 | nonsyn | smeF | A<->G | V8:1A |
| NZ_LS483377.1 | 3822369 | nonsyn | smeF | A<->G | V8:1A |
| NZ_LS483377.1 | 3822436 | stopsyn | smeE | C<->T | X3122:1X |
| NZ_LS483377.1 | 3822436 | stopsyn | smeE | C<->T | X3122:1X |
| NZ_LS483377.1 | 3822441 | syn | smeE | C<->G | T3117:2T |
| NZ_LS483377.1 | 3822441 | syn | smeE | C<->G | T3117:2T |
| NZ_LS483377.1 | 3822443 | nonsyn | smeE | T<->A | T3115:0S |
| NZ_LS483377.1 | 3822443 | nonsyn | smeE | T<->A | T3115:0S |
| NZ_LS483377.1 | 3822474 | syn | smeE | C<->G | V3084:2V |
| NZ_LS483377.1 | 3822474 | syn | smeE | C<->G | V3084:2V |
| NZ_LS483377.1 | 3822501 | syn | smeE | A<->G | G3057:2G |
| NZ_LS483377.1 | 3822501 | syn | smeE | A<->G | G3057:2G |
| NZ_LS483377.1 | 3822567 | syn | smeE | A<->G | G2991:2G |
| NZ_LS483377.1 | 3822567 | syn | smeE | A<->G | G2991:2G |
| NZ_LS483377.1 | 3822612 | syn | smeE | G<->A | A2946:2A |
| NZ_LS483377.1 | 3822612 | syn | smeE | G<->A | A2946:2A |
| NZ_LS483377.1 | 3822633 | syn | smeE | A<->C | P2925:2P |
| NZ_LS483377.1 | 3822633 | syn | smeE | A<->C | P2925:2P |
| NZ_LS483377.1 | 3822636 | syn | smeE | A<->G | R2922:2R |
| NZ_LS483377.1 | 3822636 | syn | smeE | A<->G | R2922:2R |
| NZ_LS483377.1 | 3822669 | syn | smeE | T<->C | E2889:2E |
| NZ_LS483377.1 | 3822669 | syn | smeE | T<->C | E2889:2E |
| NZ_LS483377.1 | 3822680 | nonsyn | smeE | C<->T | G2878:0S |
| NZ_LS483377.1 | 3822680 | nonsyn | smeE | C<->T | G2878:0S |
| NZ_LS483377.1 | 3822681 | syn | smeE | T<->G | A2877:2A |
| NZ_LS483377.1 | 3822681 | syn | smeE | T<->G | A2877:2A |
| NZ_LS483377.1 | 3822699 | syn | smeE | C<->T | E2859:2E |
| NZ_LS483377.1 | 3822699 | syn | smeE | C<->T | E2859:2E |
| NZ_LS483377.1 | 3822714 | syn | smeE | C<->G | V2844:2V |
| NZ_LS483377.1 | 3822714 | syn | smeE | C<->G | V2844:2V |
| NZ_LS483377.1 | 3822780 | syn | smeE | A<->G | D2778:2D |
| NZ_LS483377.1 | 3822780 | syn | smeE | A<->G | D2778:2D |
| NZ_LS483377.1 | 3822804 | syn | smeE | A<->G | N2754:2N |
| NZ_LS483377.1 | 3822804 | syn | smeE | A<->G | N2754:2N |
| NZ_LS483377.1 | 3822813 | syn | smeE | C<->T | V2745:2V |
| NZ_LS483377.1 | 3822813 | syn | smeE | C<->T | V2745:2V |
| NZ_LS483377.1 | 3822840 | syn | smeE | C<->G | A2718:2A |
| NZ_LS483377.1 | 3822840 | syn | smeE | C<->G | A2718:2A |
| NZ_LS483377.1 | 3822852 | syn | smeE | T<->G | A2706:2A |
| NZ_LS483377.1 | 3822852 | syn | smeE | T<->G | A2706:2A |
| NZ_LS483377.1 | 3822876 | syn | smeE | G<->A | Y2682:2Y |
| NZ_LS483377.1 | 3822876 | syn | smeE | G<->A | Y2682:2Y |
| NZ_LS483377.1 | 3822900 | syn | smeE | T<->C | V2658:2V |
| NZ_LS483377.1 | 3822900 | syn | smeE | T<->C | V2658:2V |
| NZ_LS483377.1 | 3822915 | syn | smeE | G<->C | L2643:2L |
| NZ_LS483377.1 | 3822915 | syn | smeE | G<->C | L2643:2L |
| NZ_LS483377.1 | 3822930 | syn | smeE | T<->C | P2628:2P |
| NZ_LS483377.1 | 3822930 | syn | smeE | T<->C | P2628:2P |
| NZ_LS483377.1 | 3822954 | syn | smeE | C<->T | E2604:2E |
| NZ_LS483377.1 | 3822954 | syn | smeE | C<->T | E2604:2E |
| NZ_LS483377.1 | 3822963 | syn | smeE | C<->G | S2595:2S |
| NZ_LS483377.1 | 3822963 | syn | smeE | C<->G | S2595:2S |
| NZ_LS483377.1 | 3823044 | syn | smeE | T<->C | A2514:2A |
| NZ_LS483377.1 | 3823044 | syn | smeE | T<->C | A2514:2A |
| NZ_LS483377.1 | 3823074 | syn | smeE | T<->C | E2484:2E |
| NZ_LS483377.1 | 3823074 | syn | smeE | T<->C | E2484:2E |
| NZ_LS483377.1 | 3823080 | syn | smeE | G<->C | A2478:2A |
| NZ_LS483377.1 | 3823080 | syn | smeE | G<->C | A2478:2A |
| NZ_LS483377.1 | 3823101 | syn | smeE | T<->C | E2457:2E |
| NZ_LS483377.1 | 3823101 | syn | smeE | T<->C | E2457:2E |
| NZ_LS483377.1 | 3823122 | syn | smeE | A<->G | D2436:2D |
| NZ_LS483377.1 | 3823122 | syn | smeE | A<->G | D2436:2D |
| NZ_LS483377.1 | 3823135 | nonsyn | smeE | G<->C | T2423:1S |
| NZ_LS483377.1 | 3823135 | nonsyn | smeE | G<->C | T2423:1S |
| NZ_LS483377.1 | 3823137 | syn | smeE | A<->G | A2421:2A |
| NZ_LS483377.1 | 3823137 | syn | smeE | A<->G | A2421:2A |
| NZ_LS483377.1 | 3823143 | syn | smeE | G<->C | A2415:2A |
| NZ_LS483377.1 | 3823143 | syn | smeE | G<->C | A2415:2A |
| NZ_LS483377.1 | 3823146 | syn | smeE | A<->G | S2412:2S |
| NZ_LS483377.1 | 3823146 | syn | smeE | A<->G | S2412:2S |
| NZ_LS483377.1 | 3823161 | syn | smeE | T<->C | E2397:2E |
| NZ_LS483377.1 | 3823161 | syn | smeE | T<->C | E2397:2E |
| NZ_LS483377.1 | 3823164 | syn | smeE | A<->G | G2394:2G |
| NZ_LS483377.1 | 3823164 | syn | smeE | A<->G | G2394:2G |
| NZ_LS483377.1 | 3823182 | syn | smeE | G<->C | S2376:2S |
| NZ_LS483377.1 | 3823182 | syn | smeE | G<->C | S2376:2S |
| NZ_LS483377.1 | 3823191 | syn | smeE | A<->G | D2367:2D |
| NZ_LS483377.1 | 3823191 | syn | smeE | A<->G | D2367:2D |
| NZ_LS483377.1 | 3823257 | syn | smeE | A<->G | R2301:2R |
| NZ_LS483377.1 | 3823257 | syn | smeE | A<->G | R2301:2R |
| NZ_LS483377.1 | 3823272 | syn | smeE | G<->A | D2286:2D |
| NZ_LS483377.1 | 3823272 | syn | smeE | G<->A | D2286:2D |
| NZ_LS483377.1 | 3823284 | syn | smeE | G<->A | S2274:2S |
| NZ_LS483377.1 | 3823284 | syn | smeE | G<->A | S2274:2S |
| NZ_LS483377.1 | 3823326 | syn | smeE | T<->C | S2232:2S |
| NZ_LS483377.1 | 3823326 | syn | smeE | T<->C | S2232:2S |
| NZ_LS483377.1 | 3823356 | syn | smeE | G<->A | D2202:2D |
| NZ_LS483377.1 | 3823356 | syn | smeE | G<->A | D2202:2D |
| NZ_LS483377.1 | 3823377 | syn | smeE | A<->C | P2181:2P |
| NZ_LS483377.1 | 3823377 | syn | smeE | A<->C | P2181:2P |
| NZ_LS483377.1 | 3823386 | syn | smeE | C<->T | E2172:2E |
| NZ_LS483377.1 | 3823386 | syn | smeE | C<->T | E2172:2E |
| NZ_LS483377.1 | 3823401 | syn | smeE | G<->A | R2157:2R |
| NZ_LS483377.1 | 3823401 | syn | smeE | G<->A | R2157:2R |
| NZ_LS483377.1 | 3823428 | syn | smeE | G<->T | A2130:2A |
| NZ_LS483377.1 | 3823428 | syn | smeE | G<->T | A2130:2A |
| NZ_LS483377.1 | 3823437 | syn | smeE | G<->C | G2121:2G |
| NZ_LS483377.1 | 3823437 | syn | smeE | G<->C | G2121:2G |
| NZ_LS483377.1 | 3823440 | syn | smeE | G<->C | L2118:2L |
| NZ_LS483377.1 | 3823440 | syn | smeE | G<->C | L2118:2L |
| NZ_LS483377.1 | 3823467 | syn | smeE | T<->G | A2091:2A |
| NZ_LS483377.1 | 3823467 | syn | smeE | T<->G | A2091:2A |
| NZ_LS483377.1 | 3823524 | syn | smeE | G<->A | G2034:2G |
| NZ_LS483377.1 | 3823524 | syn | smeE | G<->A | G2034:2G |
| NZ_LS483377.1 | 3823536 | syn | smeE | G<->A | I2022:2I |
| NZ_LS483377.1 | 3823536 | syn | smeE | G<->A | I2022:2I |
| NZ_LS483377.1 | 3823587 | syn | smeE | G<->T | A1971:2A |
| NZ_LS483377.1 | 3823587 | syn | smeE | G<->T | A1971:2A |
| NZ_LS483377.1 | 3823602 | syn | smeE | T<->C | G1956:2G |
| NZ_LS483377.1 | 3823602 | syn | smeE | T<->C | G1956:2G |
| NZ_LS483377.1 | 3823623 | syn | smeE | A<->G | N1935:2N |
| NZ_LS483377.1 | 3823623 | syn | smeE | A<->G | N1935:2N |
| NZ_LS483377.1 | 3823628 | nonsyn | smeE | T<->C | N1930:0D |
| NZ_LS483377.1 | 3823628 | nonsyn | smeE | T<->C | N1930:0D |
| NZ_LS483377.1 | 3823665 | syn | smeE | C<->G | A1893:2A |
| NZ_LS483377.1 | 3823665 | syn | smeE | C<->G | A1893:2A |
| NZ_LS483377.1 | 3823674 | syn | smeE | G<->C | A1884:2A |
| NZ_LS483377.1 | 3823674 | syn | smeE | G<->C | A1884:2A |
| NZ_LS483377.1 | 3823683 | syn | smeE | G<->T | G1875:2G |
| NZ_LS483377.1 | 3823683 | syn | smeE | G<->T | G1875:2G |
| NZ_LS483377.1 | 3823725 | nonsyn | smeE | G<->T | D1833:2E |
| NZ_LS483377.1 | 3823725 | nonsyn | smeE | G<->T | D1833:2E |
| NZ_LS483377.1 | 3823772 | nonsyn | smeE | C<->T | A1786:0T |
| NZ_LS483377.1 | 3823772 | nonsyn | smeE | C<->T | A1786:0T |
| NZ_LS483377.1 | 3823788 | syn | smeE | A<->G | R1770:2R |
| NZ_LS483377.1 | 3823788 | syn | smeE | A<->G | R1770:2R |
| NZ_LS483377.1 | 3823791 | syn | smeE | T<->C | E1767:2E |
| NZ_LS483377.1 | 3823791 | syn | smeE | T<->C | E1767:2E |
| NZ_LS483377.1 | 3823806 | syn | smeE | C<->G | V1752:2V |
| NZ_LS483377.1 | 3823806 | syn | smeE | C<->G | V1752:2V |
| NZ_LS483377.1 | 3823812 | syn | smeE | G<->T | A1746:2A |
| NZ_LS483377.1 | 3823812 | syn | smeE | G<->T | A1746:2A |
| NZ_LS483377.1 | 3823818 | syn | smeE | T<->C | V1740:2V |
| NZ_LS483377.1 | 3823818 | syn | smeE | T<->C | V1740:2V |
| NZ_LS483377.1 | 3823832 | syn | smeE | A<->G | L1726:0L |
| NZ_LS483377.1 | 3823832 | syn | smeE | A<->G | L1726:0L |
| NZ_LS483377.1 | 3823845 | syn | smeE | T<->C | E1713:2E |
| NZ_LS483377.1 | 3823845 | syn | smeE | T<->C | E1713:2E |
| NZ_LS483377.1 | 3823860 | syn | smeE | T<->C | S1698:2S |
| NZ_LS483377.1 | 3823860 | syn | smeE | T<->C | S1698:2S |
| NZ_LS483377.1 | 3823883 | syn | smeE | A<->G | L1675:0L |
| NZ_LS483377.1 | 3823883 | syn | smeE | A<->G | L1675:0L |
| NZ_LS483377.1 | 3823926 | syn | smeE | G<->A | R1632:2R |
| NZ_LS483377.1 | 3823926 | syn | smeE | G<->A | R1632:2R |
| NZ_LS483377.1 | 3823935 | syn | smeE | G<->A | R1623:2R |
| NZ_LS483377.1 | 3823935 | syn | smeE | G<->A | R1623:2R |
| NZ_LS483377.1 | 3823938 | syn | smeE | A<->G | H1620:2H |
| NZ_LS483377.1 | 3823938 | syn | smeE | A<->G | H1620:2H |
| NZ_LS483377.1 | 3823989 | syn | smeE | T<->A | G1569:2G |
| NZ_LS483377.1 | 3823989 | syn | smeE | T<->A | G1569:2G |
| NZ_LS483377.1 | 3824001 | syn | smeE | G<->A | G1557:2G |
| NZ_LS483377.1 | 3824001 | syn | smeE | G<->A | G1557:2G |
| NZ_LS483377.1 | 3824004 | syn | smeE | A<->G | N1554:2N |
| NZ_LS483377.1 | 3824004 | syn | smeE | A<->G | N1554:2N |
| NZ_LS483377.1 | 3824013 | syn | smeE | G<->A | R1545:2R |
| NZ_LS483377.1 | 3824013 | syn | smeE | G<->A | R1545:2R |
| NZ_LS483377.1 | 3824028 | syn | smeE | G<->C | R1530:2R |
| NZ_LS483377.1 | 3824028 | syn | smeE | G<->C | R1530:2R |
| NZ_LS483377.1 | 3824029 | nonsyn | smeE | C<->T | R1529:1H |
| NZ_LS483377.1 | 3824029 | nonsyn | smeE | C<->T | R1529:1H |
| NZ_LS483377.1 | 3824030 | nonsyn | smeE | G<->T | R1528:0S |
| NZ_LS483377.1 | 3824030 | nonsyn | smeE | G<->T | R1528:0S |
| NZ_LS483377.1 | 3824034 | syn | smeE | G<->C | A1524:2A |
| NZ_LS483377.1 | 3824034 | syn | smeE | G<->C | A1524:2A |
| NZ_LS483377.1 | 3824049 | syn | smeE | A<->G | G1509:2G |
| NZ_LS483377.1 | 3824049 | syn | smeE | A<->G | G1509:2G |
| NZ_LS483377.1 | 3824085 | syn | smeE | T<->C | A1473:2A |
| NZ_LS483377.1 | 3824085 | syn | smeE | T<->C | A1473:2A |
| NZ_LS483377.1 | 3824106 | syn | smeE | G<->C | V1452:2V |
| NZ_LS483377.1 | 3824106 | syn | smeE | G<->C | V1452:2V |
| NZ_LS483377.1 | 3824121 | syn | smeE | T<->C | A1437:2A |
| NZ_LS483377.1 | 3824121 | syn | smeE | T<->C | A1437:2A |
| NZ_LS483377.1 | 3824178 | syn | smeE | A<->G | S1380:2S |
| NZ_LS483377.1 | 3824178 | syn | smeE | A<->G | S1380:2S |
| NZ_LS483377.1 | 3824226 | syn | smeE | G<->A | G1332:2G |
| NZ_LS483377.1 | 3824226 | syn | smeE | G<->A | G1332:2G |
| NZ_LS483377.1 | 3824250 | syn | smeE | G<->C | G1308:2G |
| NZ_LS483377.1 | 3824250 | syn | smeE | G<->C | G1308:2G |
| NZ_LS483377.1 | 3824271 | syn | smeE | C<->T | E1287:2E |
| NZ_LS483377.1 | 3824271 | syn | smeE | C<->T | E1287:2E |
| NZ_LS483377.1 | 3824319 | syn | smeE | A<->C | V1239:2V |
| NZ_LS483377.1 | 3824319 | syn | smeE | A<->C | V1239:2V |
| NZ_LS483377.1 | 3824358 | syn | smeE | A<->C | L1200:2L |
| NZ_LS483377.1 | 3824358 | syn | smeE | A<->C | L1200:2L |
| NZ_LS483377.1 | 3824400 | syn | smeE | G<->C | L1158:2L |
| NZ_LS483377.1 | 3824400 | syn | smeE | G<->C | L1158:2L |
| NZ_LS483377.1 | 3824406 | syn | smeE | G<->C | A1152:2A |
| NZ_LS483377.1 | 3824406 | syn | smeE | G<->C | A1152:2A |
| NZ_LS483377.1 | 3824585 | nonsyn | smeE | C<->T | V973:0I |
| NZ_LS483377.1 | 3824585 | nonsyn | smeE | C<->T | V973:0I |
| NZ_LS483377.1 | 3824625 | syn | smeE | T<->C | E933:2E |
| NZ_LS483377.1 | 3824625 | syn | smeE | T<->C | E933:2E |
| NZ_LS483377.1 | 3824627 | nonsyn | smeE | C<->G | E931:0Q |
| NZ_LS483377.1 | 3824627 | nonsyn | smeE | C<->G | E931:0Q |
| NZ_LS483377.1 | 3824639 | nonsyn | smeE | T<->C | N919:0D |
| NZ_LS483377.1 | 3824639 | nonsyn | smeE | T<->C | N919:0D |
| NZ_LS483377.1 | 3824646 | syn | smeE | A<->G | A912:2A |
| NZ_LS483377.1 | 3824646 | syn | smeE | A<->G | A912:2A |
| NZ_LS483377.1 | 3824649 | syn | smeE | A<->C | A909:2A |
| NZ_LS483377.1 | 3824649 | syn | smeE | A<->C | A909:2A |
| NZ_LS483377.1 | 3824655 | syn | smeE | A<->G | D903:2D |
| NZ_LS483377.1 | 3824655 | syn | smeE | A<->G | D903:2D |
| NZ_LS483377.1 | 3824685 | syn | smeE | C<->G | V873:2V |
| NZ_LS483377.1 | 3824685 | syn | smeE | C<->G | V873:2V |
| NZ_LS483377.1 | 3824751 | syn | smeE | C<->T | E807:2E |
| NZ_LS483377.1 | 3824751 | syn | smeE | C<->T | E807:2E |
| NZ_LS483377.1 | 3824769 | syn | smeE | C<->A | G789:2G |
| NZ_LS483377.1 | 3824769 | syn | smeE | C<->A | G789:2G |
| NZ_LS483377.1 | 3824781 | syn | smeE | C<->T | E777:2E |
| NZ_LS483377.1 | 3824781 | syn | smeE | C<->T | E777:2E |
| NZ_LS483377.1 | 3824885 | nonsyn | smeE | T<->C | I673:0V |
| NZ_LS483377.1 | 3824885 | nonsyn | smeE | T<->C | I673:0V |
| NZ_LS483377.1 | 3824892 | syn | smeE | T<->C | A666:2A |
| NZ_LS483377.1 | 3824892 | syn | smeE | T<->C | A666:2A |
| NZ_LS483377.1 | 3824922 | syn | smeE | C<->G | A636:2A |
| NZ_LS483377.1 | 3824922 | syn | smeE | C<->G | A636:2A |
| NZ_LS483377.1 | 3824931 | syn | smeE | T<->C | A627:2A |
| NZ_LS483377.1 | 3824931 | syn | smeE | T<->C | A627:2A |
| NZ_LS483377.1 | 3824937 | nonsyn | smeE | G<->C | I621:2M |
| NZ_LS483377.1 | 3824937 | nonsyn | smeE | G<->C | I621:2M |
| NZ_LS483377.1 | 3824939 | nonsyn | smeE | T<->C | I619:0V |
| NZ_LS483377.1 | 3824939 | nonsyn | smeE | T<->C | I619:0V |
| NZ_LS483377.1 | 3824946 | syn | smeE | G<->C | T612:2T |
| NZ_LS483377.1 | 3824946 | syn | smeE | G<->C | T612:2T |
| NZ_LS483377.1 | 3824955 | syn | smeE | C<->G | A603:2A |
| NZ_LS483377.1 | 3824955 | syn | smeE | C<->G | A603:2A |
| NZ_LS483377.1 | 3824970 | syn | smeE | A<->G | Y588:2Y |
| NZ_LS483377.1 | 3824970 | syn | smeE | A<->G | Y588:2Y |
| NZ_LS483377.1 | 3824976 | syn | smeE | A<->G | H582:2H |
| NZ_LS483377.1 | 3824976 | syn | smeE | A<->G | H582:2H |
| NZ_LS483377.1 | 3824991 | syn | smeE | A<->G | D567:2D |
| NZ_LS483377.1 | 3824991 | syn | smeE | A<->G | D567:2D |
| NZ_LS483377.1 | 3825063 | syn | smeE | G<->A | R495:2R |
| NZ_LS483377.1 | 3825063 | syn | smeE | G<->A | R495:2R |
| NZ_LS483377.1 | 3825066 | syn | smeE | A<->G | D492:2D |
| NZ_LS483377.1 | 3825066 | syn | smeE | A<->G | D492:2D |
| NZ_LS483377.1 | 3825075 | syn | smeE | A<->G | N483:2N |
| NZ_LS483377.1 | 3825075 | syn | smeE | A<->G | N483:2N |
| NZ_LS483377.1 | 3825108 | syn | smeE | A<->G | D450:2D |
| NZ_LS483377.1 | 3825108 | syn | smeE | A<->G | D450:2D |
| NZ_LS483377.1 | 3825120 | syn | smeE | G<->A | N438:2N |
| NZ_LS483377.1 | 3825120 | syn | smeE | G<->A | N438:2N |
| NZ_LS483377.1 | 3825192 | syn | smeE | A<->T | V366:2V |
| NZ_LS483377.1 | 3825192 | syn | smeE | A<->T | V366:2V |
| NZ_LS483377.1 | 3825411 | syn | smeE | G<->A | Y147:2Y |
| NZ_LS483377.1 | 3825411 | syn | smeE | G<->A | Y147:2Y |
| NZ_LS483377.1 | 3825522 | syn | smeE | C<->T | A36:2A |
| NZ_LS483377.1 | 3825522 | syn | smeE | C<->T | A36:2A |
| NZ_LS483377.1 | 3825525 | syn | smeE | G<->A | F33:2F |
| NZ_LS483377.1 | 3825525 | syn | smeE | G<->A | F33:2F |
| NZ_LS483377.1 | 3825609 | syn | smeD | C<->T | A1146:2A |
| NZ_LS483377.1 | 3825609 | syn | smeD | C<->T | A1146:2A |
| NZ_LS483377.1 | 3825612 | syn | smeD | T<->C | A1143:2A |
| NZ_LS483377.1 | 3825612 | syn | smeD | T<->C | A1143:2A |
| NZ_LS483377.1 | 3825627 | syn | smeD | A<->T | A1128:2A |
| NZ_LS483377.1 | 3825627 | syn | smeD | A<->T | A1128:2A |
| NZ_LS483377.1 | 3825690 | syn | smeD | G<->A | I1065:2I |
| NZ_LS483377.1 | 3825690 | syn | smeD | G<->A | I1065:2I |
| NZ_LS483377.1 | 3825714 | syn | smeD | A<->G | G1041:2G |
| NZ_LS483377.1 | 3825714 | syn | smeD | A<->G | G1041:2G |
| NZ_LS483377.1 | 3825720 | nonsyn | smeD | C<->A | E1035:2D |
| NZ_LS483377.1 | 3825720 | nonsyn | smeD | C<->A | E1035:2D |
| NZ_LS483377.1 | 3825790 | nonsyn | smeD | C<->T | G965:1D |
| NZ_LS483377.1 | 3825790 | nonsyn | smeD | C<->T | G965:1D |
| NZ_LS483377.1 | 3825804 | syn | smeD | G<->C | T951:2T |
| NZ_LS483377.1 | 3825804 | syn | smeD | G<->C | T951:2T |
| NZ_LS483377.1 | 3825825 | syn | smeD | G<->A | R930:2R |
| NZ_LS483377.1 | 3825825 | syn | smeD | G<->A | R930:2R |
| NZ_LS483377.1 | 3825846 | syn | smeD | T<->C | V909:2V |
| NZ_LS483377.1 | 3825846 | syn | smeD | T<->C | V909:2V |
| NZ_LS483377.1 | 3825888 | syn | smeD | G<->A | R867:2R |
| NZ_LS483377.1 | 3825888 | syn | smeD | G<->A | R867:2R |
| NZ_LS483377.1 | 3825897 | syn | smeD | G<->C | T858:2T |
| NZ_LS483377.1 | 3825897 | syn | smeD | G<->C | T858:2T |
| NZ_LS483377.1 | 3825898 | nonsyn | smeD | G<->A | T857:1I |
| NZ_LS483377.1 | 3825898 | nonsyn | smeD | G<->A | T857:1I |
| NZ_LS483377.1 | 3825921 | syn | smeD | C<->G | P834:2P |
| NZ_LS483377.1 | 3825921 | syn | smeD | C<->G | P834:2P |
| NZ_LS483377.1 | 3825963 | syn | smeD | T<->C | P792:2P |
| NZ_LS483377.1 | 3825963 | syn | smeD | T<->C | P792:2P |
| NZ_LS483377.1 | 3825975 | syn | smeD | C<->G | V780:2V |
| NZ_LS483377.1 | 3825975 | syn | smeD | C<->G | V780:2V |
| NZ_LS483377.1 | 3825978 | syn | smeD | T<->C | E777:2E |
| NZ_LS483377.1 | 3825978 | syn | smeD | T<->C | E777:2E |
| NZ_LS483377.1 | 3825981 | syn | smeD | A<->G | S774:2S |
| NZ_LS483377.1 | 3825981 | syn | smeD | A<->G | S774:2S |
| NZ_LS483377.1 | 3825984 | syn | smeD | G<->A | F771:2F |
| NZ_LS483377.1 | 3825984 | syn | smeD | G<->A | F771:2F |
| NZ_LS483377.1 | 3826047 | syn | smeD | C<->G | T708:2T |
| NZ_LS483377.1 | 3826047 | syn | smeD | C<->G | T708:2T |
| NZ_LS483377.1 | 3826074 | syn | smeD | A<->G | A681:2A |
| NZ_LS483377.1 | 3826074 | syn | smeD | A<->G | A681:2A |
| NZ_LS483377.1 | 3826086 | syn | smeD | G<->A | R669:2R |
| NZ_LS483377.1 | 3826086 | syn | smeD | G<->A | R669:2R |
| NZ_LS483377.1 | 3826122 | syn | smeD | A<->G | D633:2D |
| NZ_LS483377.1 | 3826122 | syn | smeD | A<->G | D633:2D |
| NZ_LS483377.1 | 3826125 | syn | smeD | G<->C | V630:2V |
| NZ_LS483377.1 | 3826125 | syn | smeD | G<->C | V630:2V |
| NZ_LS483377.1 | 3826128 | syn | smeD | G<->A | Y627:2Y |
| NZ_LS483377.1 | 3826128 | syn | smeD | G<->A | Y627:2Y |
| NZ_LS483377.1 | 3826161 | syn | smeD | T<->C | A594:2A |
| NZ_LS483377.1 | 3826161 | syn | smeD | T<->C | A594:2A |
| NZ_LS483377.1 | 3826164 | syn | smeD | A<->G | N591:2N |
| NZ_LS483377.1 | 3826164 | syn | smeD | A<->G | N591:2N |
| NZ_LS483377.1 | 3826200 | syn | smeD | A<->G | V555:2V |
| NZ_LS483377.1 | 3826200 | syn | smeD | A<->G | V555:2V |
| NZ_LS483377.1 | 3826248 | syn | smeD | A<->G | Y507:2Y |
| NZ_LS483377.1 | 3826248 | syn | smeD | A<->G | Y507:2Y |
| NZ_LS483377.1 | 3826285 | nonsyn | smeD | C<->T | R470:1K |
| NZ_LS483377.1 | 3826285 | nonsyn | smeD | C<->T | R470:1K |
| NZ_LS483377.1 | 3826398 | syn | smeD | G<->C | R357:2R |
| NZ_LS483377.1 | 3826398 | syn | smeD | G<->C | R357:2R |
| NZ_LS483377.1 | 3826401 | syn | smeD | C<->G | A354:2A |
| NZ_LS483377.1 | 3826401 | syn | smeD | C<->G | A354:2A |
| NZ_LS483377.1 | 3826413 | syn | smeD | C<->G | T342:2T |
| NZ_LS483377.1 | 3826413 | syn | smeD | C<->G | T342:2T |
| NZ_LS483377.1 | 3826447 | nonsyn | smeD | C<->T | S308:1N |
| NZ_LS483377.1 | 3826447 | nonsyn | smeD | C<->T | S308:1N |
| NZ_LS483377.1 | 3826476 | nonsyn | smeD | C<->A | E279:2D |
| NZ_LS483377.1 | 3826476 | nonsyn | smeD | C<->A | E279:2D |
| NZ_LS483377.1 | 3826481 | nonsyn | smeD | T<->G | I274:0L |
| NZ_LS483377.1 | 3826481 | nonsyn | smeD | T<->G | I274:0L |
| NZ_LS483377.1 | 3826488 | syn | smeD | G<->C | L267:2L |
| NZ_LS483377.1 | 3826488 | syn | smeD | G<->C | L267:2L |
| NZ_LS483377.1 | 3826497 | syn | smeD | G<->A | G258:2G |
| NZ_LS483377.1 | 3826497 | syn | smeD | G<->A | G258:2G |
| NZ_LS483377.1 | 3826518 | syn | smeD | T<->C | E237:2E |
| NZ_LS483377.1 | 3826518 | syn | smeD | T<->C | E237:2E |
| NZ_LS483377.1 | 3826548 | syn | smeD | G<->A | N207:2N |
| NZ_LS483377.1 | 3826548 | syn | smeD | G<->A | N207:2N |
| NZ_LS483377.1 | 3826572 | syn | smeD | G<->C | V183:2V |
| NZ_LS483377.1 | 3826572 | syn | smeD | G<->C | V183:2V |
| NZ_LS483377.1 | 3826584 | syn | smeD | G<->A | N171:2N |
| NZ_LS483377.1 | 3826584 | syn | smeD | G<->A | N171:2N |
| NZ_LS483377.1 | 3826596 | syn | smeD | C<->G | P159:2P |
| NZ_LS483377.1 | 3826596 | syn | smeD | C<->G | P159:2P |
| NZ_LS483377.1 | 3826665 | syn | smeD | C<->T | E90:2E |
| NZ_LS483377.1 | 3826665 | syn | smeD | C<->T | E90:2E |
| NZ_LS483377.1 | 3826689 | syn | smeD | G<->A | C66:2C |
| NZ_LS483377.1 | 3826689 | syn | smeD | G<->A | C66:2C |
| NZ_LS483377.1 | 3826703 | nonsyn | smeD | C<->T | A52:0T |
| NZ_LS483377.1 | 3826703 | nonsyn | smeD | C<->T | A52:0T |
| NZ_LS483377.1 | 3826704 | syn | smeD | T<->C | A51:2A |
| NZ_LS483377.1 | 3826704 | syn | smeD | T<->C | A51:2A |
| NZ_LS483377.1 | 3826725 | syn | smeD | T<->C | A30:2A |
| NZ_LS483377.1 | 3826725 | syn | smeD | T<->C | A30:2A |
| NZ_LS483377.1 | 3826728 | syn | smeD | A<->G | F27:2F |
| NZ_LS483377.1 | 3826728 | syn | smeD | A<->G | F27:2F |

Table S4 SNPs possessed by WJ_97 compared to WJ_4a, WJ_14a, WJ_18a

| Chromosomes | Position | Type of SNP | Gene | Mutation mode | Amino acid mutations |
| --- | --- | --- | --- | --- | --- |
| NZ_LS483377.1 | 3822365 | Deletion | smeF | A | - |
| NZ_LS483377.1 | 3822365 | Deletion | smeF | A | - |
| NZ_LS483377.1 | 3822365 | Deletion | smeF | A | - |
| NZ_LS483377.1 | 3822365 | Deletion | smeF | A | - |
| NZ_LS483377.1 | 3822365 | Deletion | smeF | A | - |
| NZ_LS483377.1 | 3822365 | Deletion | smeF | A | - |
| NZ_LS483377.1 | 3822365 | Deletion | smeF | A | - |
| NZ_LS483377.1 | 3822365 | Deletion | smeF | A | - |
| NZ_LS483377.1 | 1364937 | syn | smeR | T<->C | T21:2T |
| NZ_LS483377.1 | 1365043 | nonsyn | smeR | G<->A | G127:0R |
| NZ_LS483377.1 | 1365048 | syn | smeR | G<->A | E132:2E |
| NZ_LS483377.1 | 1365060 | syn | smeR | G<->T | R144:2R |
| NZ_LS483377.1 | 1365074 | nonsyn | smeR | A<->G | D158:1G |
| NZ_LS483377.1 | 1365075 | syn | smeR | T<->C | D159:2D |
| NZ_LS483377.1 | 1365081 | syn | smeR | C<->T | D165:2D |
| NZ_LS483377.1 | 1365082 | nonsyn | smeR | G<->C | V166:0L |
| NZ_LS483377.1 | 1365085 | nonsyn | smeR | G<->C | V169:0L |
| NZ_LS483377.1 | 1365108 | syn | smeR | C<->G | P192:2P |
| NZ_LS483377.1 | 1365111 | syn | smeR | C<->T | D195:2D |
| NZ_LS483377.1 | 1365153 | syn | smeR | C<->G | P237:2P |
| NZ_LS483377.1 | 1365156 | syn | smeR | G<->C | G240:2G |
| NZ_LS483377.1 | 1365162 | syn | smeR | G<->T | A246:2A |
| NZ_LS483377.1 | 1365234 | syn | smeR | C<->G | G318:2G |
| NZ_LS483377.1 | 1365309 | syn | smeR | G<->C | A393:2A |
| NZ_LS483377.1 | 1365316 | nonsyn | smeR | G<->C | V400:0L |
| NZ_LS483377.1 | 1365328 | nonsyn | smeR | C<->G | P412:0A |
| NZ_LS483377.1 | 1365329 | nonsyn | smeR | C<->G | P413:1R |
| NZ_LS483377.1 | 1365339 | nonsyn | smeR | T<->A | D423:2E |
| NZ_LS483377.1 | 1365354 | syn | smeR | A<->G | E438:2E |
| NZ_LS483377.1 | 1365357 | syn | smeR | T<->C | H441:2H |
| NZ_LS483377.1 | 1365381 | nonsyn | smeR | A<->C | E465:2D |
| NZ_LS483377.1 | 1365387 | syn | smeR | G<->C | R471:2R |
| NZ_LS483377.1 | 1365400 | nonsyn | smeR | C<->A | R484:0S |
| NZ_LS483377.1 | 1365432 | syn | smeR | G<->A | E516:2E |
| NZ_LS483377.1 | 1365450 | syn | smeR | G<->A | A534:2A |
| NZ_LS483377.1 | 1365459 | syn | smeR | G<->A | Q543:2Q |
| NZ_LS483377.1 | 1365486 | syn | smeR | C<->A | A570:2A |
| NZ_LS483377.1 | 1365498 | syn | smeR | C<->T | D582:2D |
| NZ_LS483377.1 | 1365501 | syn | smeR | T<->C | R585:2R |
| NZ_LS483377.1 | 1365534 | syn | smeR | C<->T | R618:2R |
| NZ_LS483377.1 | 1365543 | syn | smeR | T<->C | D627:2D |
| NZ_LS483377.1 | 1365576 | syn | smeR | C<->T | R660:2R |
| NZ_LS483377.1 | 1365591 | syn | smeR | C<->T | S675:2S |
| NZ_LS483377.1 | 1365597 | syn | smeR | C<->T | D681:2D |
| NZ_LS483377.1 | 1365621 | syn | smeR | T<->C | L705:2L |
| NZ_LS483377.1 | 1753461 | syn | adeF | A<->G | P90:2P |
| NZ_LS483377.1 | 1753509 | syn | adeF | C<->G | V138:2V |
| NZ_LS483377.1 | 1753521 | syn | adeF | C<->G | V150:2V |
| NZ_LS483377.1 | 1753632 | syn | adeF | C<->T | D261:2D |
| NZ_LS483377.1 | 1753902 | syn | adeF | G<->A | A531:2A |
| NZ_LS483377.1 | 1753923 | syn | adeF | C<->T | D552:2D |
| NZ_LS483377.1 | 1754022 | syn | adeF | G<->C | V651:2V |
| NZ_LS483377.1 | 1754056 | nonsyn | adeF | G<->A | D685:0N |
| NZ_LS483377.1 | 1754062 | nonsyn | adeF | A<->C | K691:0Q |
| NZ_LS483377.1 | 1754082 | syn | adeF | T<->C | N711:2N |
| NZ_LS483377.1 | 1754140 | nonsyn | adeF | A<->G | T769:0A |
| NZ_LS483377.1 | 1754141 | nonsyn | adeF | C<->T | T770:1I |
| NZ_LS483377.1 | 1754164 | nonsyn | adeF | T<->G | S793:0A |
| NZ_LS483377.1 | 1754193 | syn | adeF | G<->C | A822:2A |
| NZ_LS483377.1 | 1754205 | syn | adeF | C<->G | T834:2T |
| NZ_LS483377.1 | 1754247 | syn | adeF | C<->T | G876:2G |
| NZ_LS483377.1 | 1754271 | syn | adeF | C<->T | N900:2N |
| NZ_LS483377.1 | 1754274 | syn | adeF | G<->A | A903:2A |
| NZ_LS483377.1 | 1754280 | syn | adeF | A<->G | E909:2E |
| NZ_LS483377.1 | 1754358 | syn | adeF | T<->C | Y987:2Y |
| NZ_LS483377.1 | 1754370 | syn | adeF | C<->T | I999:2I |
| NZ_LS483377.1 | 1754400 | syn | adeF | C<->T | V1029:2V |
| NZ_LS483377.1 | 1754418 | syn | adeF | G<->C | A1047:2A |
| NZ_LS483377.1 | 1754425 | syn | adeF | C<->T | L1054:0L |
| NZ_LS483377.1 | 1754508 | syn | adeF | G<->C | V1137:2V |
| NZ_LS483377.1 | 1754511 | syn | adeF | T<->C | G1140:2G |
| NZ_LS483377.1 | 1754520 | syn | adeF | C<->T | A1149:2A |
| NZ_LS483377.1 | 1754568 | syn | adeF | C<->G | G1197:2G |
| NZ_LS483377.1 | 1754682 | syn | adeF | C<->T | R1311:2R |
| NZ_LS483377.1 | 1754745 | syn | adeF | G<->C | A1374:2A |
| NZ_LS483377.1 | 1754847 | syn | adeF | C<->G | A1476:2A |
| NZ_LS483377.1 | 1754862 | syn | adeF | G<->T | L1491:2L |
| NZ_LS483377.1 | 1755117 | syn | adeF | C<->T | G1746:2G |
| NZ_LS483377.1 | 1755135 | syn | adeF | T<->C | G1764:2G |
| NZ_LS483377.1 | 1755174 | syn | adeF | G<->C | V1803:2V |
| NZ_LS483377.1 | 1755198 | syn | adeF | T<->C | D1827:2D |
| NZ_LS483377.1 | 1755216 | syn | adeF | C<->T | I1845:2I |
| NZ_LS483377.1 | 1755258 | syn | adeF | C<->G | P1887:2P |
| NZ_LS483377.1 | 1755459 | syn | adeF | C<->T | G2088:2G |
| NZ_LS483377.1 | 1755469 | syn | adeF | C<->T | L2098:0L |
| NZ_LS483377.1 | 1755486 | syn | adeF | G<->A | Q2115:2Q |
| NZ_LS483377.1 | 1755570 | syn | adeF | C<->G | A2199:2A |
| NZ_LS483377.1 | 1755573 | syn | adeF | G<->A | E2202:2E |
| NZ_LS483377.1 | 1755597 | syn | adeF | A<->G | A2226:2A |
| NZ_LS483377.1 | 1755657 | syn | adeF | C<->T | Y2286:2Y |
| NZ_LS483377.1 | 1755681 | syn | adeF | C<->T | G2310:2G |
| NZ_LS483377.1 | 1755702 | syn | adeF | C<->G | A2331:2A |
| NZ_LS483377.1 | 1755849 | syn | adeF | C<->T | Y2478:2Y |
| NZ_LS483377.1 | 1755894 | syn | adeF | C<->G | S2523:2S |
| NZ_LS483377.1 | 1755911 | nonsyn | adeF | C<->A | T2540:1N |
| NZ_LS483377.1 | 1755927 | syn | adeF | C<->T | I2556:2I |
| NZ_LS483377.1 | 1755930 | syn | adeF | C<->G | A2559:2A |
| NZ_LS483377.1 | 1756005 | syn | adeF | C<->G | A2634:2A |
| NZ_LS483377.1 | 1756008 | syn | adeF | C<->G | A2637:2A |
| NZ_LS483377.1 | 1756189 | syn | adeF | C<->T | L2818:0L |
| NZ_LS483377.1 | 1756269 | syn | adeF | T<->C | V2898:2V |
| NZ_LS483377.1 | 1756287 | syn | adeF | C<->G | A2916:2A |
| NZ_LS483377.1 | 1756467 | syn | adeF | G<->C | V3096:2V |
| NZ_LS483377.1 | 1756497 | syn | adeF | T<->C | G3126:2G |
| NZ_LS483377.1 | 3820997 | syn | smeF | A<->G | G1380:2G |
| NZ_LS483377.1 | 3821017 | syn | smeF | G<->A | L1360:0L |
| NZ_LS483377.1 | 3821039 | syn | smeF | C<->G | A1338:2A |
| NZ_LS483377.1 | 3821060 | syn | smeF | G<->C | T1317:2T |
| NZ_LS483377.1 | 3821069 | syn | smeF | T<->C | A1308:2A |
| NZ_LS483377.1 | 3821090 | syn | smeF | C<->T | A1287:2A |
| NZ_LS483377.1 | 3821105 | syn | smeF | C<->G | V1272:2V |
| NZ_LS483377.1 | 3821123 | syn | smeF | C<->G | A1254:2A |
| NZ_LS483377.1 | 3821141 | syn | smeF | C<->T | S1236:2S |
| NZ_LS483377.1 | 3821168 | nonsyn | smeF | T<->A | E1209:2D |
| NZ_LS483377.1 | 3821188 | nonsyn | smeF | A<->C | S1189:0A |
| NZ_LS483377.1 | 3821201 | syn | smeF | G<->A | D1176:2D |
| NZ_LS483377.1 | 3821218 | syn | smeF | A<->G | L1159:0L |
| NZ_LS483377.1 | 3821228 | syn | smeF | A<->G | D1149:2D |
| NZ_LS483377.1 | 3821249 | syn | smeF | C<->G | V1128:2V |
| NZ_LS483377.1 | 3821258 | syn | smeF | C<->T | S1119:2S |
| NZ_LS483377.1 | 3821279 | syn | smeF | A<->C | A1098:2A |
| NZ_LS483377.1 | 3821288 | syn | smeF | A<->G | R1089:2R |
| NZ_LS483377.1 | 3821291 | syn | smeF | A<->G | D1086:2D |
| NZ_LS483377.1 | 3821297 | syn | smeF | G<->A | N1080:2N |
| NZ_LS483377.1 | 3821348 | syn | smeF | C<->G | T1029:2T |
| NZ_LS483377.1 | 3821378 | syn | smeF | C<->G | T999:2T |
| NZ_LS483377.1 | 3821381 | syn | smeF | A<->G | G996:2G |
| NZ_LS483377.1 | 3821462 | syn | smeF | G<->A | R915:2R |
| NZ_LS483377.1 | 3821471 | syn | smeF | A<->G | G906:2G |
| NZ_LS483377.1 | 3821477 | syn | smeF | A<->G | N900:2N |
| NZ_LS483377.1 | 3821489 | syn | smeF | C<->A | A888:2A |
| NZ_LS483377.1 | 3821555 | syn | smeF | G<->A | A822:2A |
| NZ_LS483377.1 | 3821573 | syn | smeF | T<->C | A804:2A |
| NZ_LS483377.1 | 3821611 | nonsyn | smeF | C<->A | A766:0S |
| NZ_LS483377.1 | 3821624 | syn | smeF | G<->A | G753:2G |
| NZ_LS483377.1 | 3821639 | syn | smeF | C<->T | A738:2A |
| NZ_LS483377.1 | 3821669 | syn | smeF | G<->C | G708:2G |
| NZ_LS483377.1 | 3821702 | syn | smeF | C<->T | E675:2E |
| NZ_LS483377.1 | 3821705 | syn | smeF | G<->C | V672:2V |
| NZ_LS483377.1 | 3821750 | syn | smeF | A<->G | R627:2R |
| NZ_LS483377.1 | 3821756 | syn | smeF | G<->A | H621:2H |
| NZ_LS483377.1 | 3821765 | syn | smeF | C<->T | E612:2E |
| NZ_LS483377.1 | 3821828 | syn | smeF | C<->T | A549:2A |
| NZ_LS483377.1 | 3821881 | syn | smeF | A<->G | L496:0L |
| NZ_LS483377.1 | 3821888 | syn | smeF | G<->A | N489:2N |
| NZ_LS483377.1 | 3821897 | syn | smeF | G<->A | N480:2N |
| NZ_LS483377.1 | 3821903 | syn | smeF | G<->C | A474:2A |
| NZ_LS483377.1 | 3821933 | syn | smeF | T<->C | E444:2E |
| NZ_LS483377.1 | 3821936 | syn | smeF | A<->G | S441:2S |
| NZ_LS483377.1 | 3821942 | syn | smeF | A<->G | N435:2N |
| NZ_LS483377.1 | 3821984 | syn | smeF | G<->A | G393:2G |
| NZ_LS483377.1 | 3821993 | syn | smeF | G<->C | A384:2A |
| NZ_LS483377.1 | 3822014 | syn | smeF | A<->G | G363:2G |
| NZ_LS483377.1 | 3822065 | syn | smeF | C<->T | V312:2V |
| NZ_LS483377.1 | 3822071 | syn | smeF | A<->G | D306:2D |
| NZ_LS483377.1 | 3822074 | syn | smeF | A<->G | A303:2A |
| NZ_LS483377.1 | 3822101 | syn | smeF | C<->T | A276:2A |
| NZ_LS483377.1 | 3822110 | syn | smeF | A<->C | V267:2V |
| NZ_LS483377.1 | 3822176 | syn | smeF | G<->T | R201:2R |
| NZ_LS483377.1 | 3822182 | syn | smeF | A<->G | D195:2D |
| NZ_LS483377.1 | 3822215 | syn | smeF | G<->A | V162:2V |
| NZ_LS483377.1 | 3822229 | nonsyn | smeF | C<->G | E148:0Q |
| NZ_LS483377.1 | 3822238 | nonsyn | smeF | C<->T | A139:0T |
| NZ_LS483377.1 | 3822263 | syn | smeF | G<->A | I114:2I |
| NZ_LS483377.1 | 3822266 | syn | smeF | C<->G | A111:2A |
| NZ_LS483377.1 | 3822272 | syn | smeF | G<->C | A105:2A |
| NZ_LS483377.1 | 3822278 | syn | smeF | A<->G | A99:2A |
| NZ_LS483377.1 | 3822320 | syn | smeF | G<->A | L57:2L |
| NZ_LS483377.1 | 3822369 | nonsyn | smeF | A<->G | V8:1A |
| NZ_LS483377.1 | 3822436 | stopsyn | smeE | C<->T | X3122:1X |
| NZ_LS483377.1 | 3822441 | syn | smeE | C<->G | T3117:2T |
| NZ_LS483377.1 | 3822443 | nonsyn | smeE | T<->A | T3115:0S |
| NZ_LS483377.1 | 3822474 | syn | smeE | C<->G | V3084:2V |
| NZ_LS483377.1 | 3822567 | syn | smeE | A<->G | G2991:2G |
| NZ_LS483377.1 | 3822612 | syn | smeE | G<->A | A2946:2A |
| NZ_LS483377.1 | 3822813 | syn | smeE | C<->T | V2745:2V |
| NZ_LS483377.1 | 3822840 | syn | smeE | C<->G | A2718:2A |
| NZ_LS483377.1 | 3822930 | syn | smeE | T<->C | P2628:2P |
| NZ_LS483377.1 | 3823122 | syn | smeE | A<->G | D2436:2D |
| NZ_LS483377.1 | 3823135 | nonsyn | smeE | G<->C | T2423:1S |
| NZ_LS483377.1 | 3823146 | syn | smeE | A<->G | S2412:2S |
| NZ_LS483377.1 | 3823182 | syn | smeE | G<->C | S2376:2S |
| NZ_LS483377.1 | 3823257 | syn | smeE | A<->G | R2301:2R |
| NZ_LS483377.1 | 3823284 | syn | smeE | G<->A | S2274:2S |
| NZ_LS483377.1 | 3823356 | syn | smeE | G<->A | D2202:2D |
| NZ_LS483377.1 | 3823386 | syn | smeE | C<->T | E2172:2E |
| NZ_LS483377.1 | 3823401 | syn | smeE | G<->A | R2157:2R |
| NZ_LS483377.1 | 3823428 | syn | smeE | G<->T | A2130:2A |
| NZ_LS483377.1 | 3823437 | syn | smeE | G<->C | G2121:2G |
| NZ_LS483377.1 | 3823440 | syn | smeE | G<->C | L2118:2L |
| NZ_LS483377.1 | 3823587 | syn | smeE | G<->T | A1971:2A |
| NZ_LS483377.1 | 3823623 | syn | smeE | A<->G | N1935:2N |
| NZ_LS483377.1 | 3823674 | syn | smeE | G<->C | A1884:2A |
| NZ_LS483377.1 | 3823683 | syn | smeE | G<->T | G1875:2G |
| NZ_LS483377.1 | 3823772 | nonsyn | smeE | C<->T | A1786:0T |
| NZ_LS483377.1 | 3823845 | syn | smeE | T<->C | E1713:2E |
| NZ_LS483377.1 | 3823926 | syn | smeE | G<->A | R1632:2R |
| NZ_LS483377.1 | 3823938 | syn | smeE | A<->G | H1620:2H |
| NZ_LS483377.1 | 3824013 | syn | smeE | G<->A | R1545:2R |
| NZ_LS483377.1 | 3824028 | syn | smeE | G<->C | R1530:2R |
| NZ_LS483377.1 | 3824029 | nonsyn | smeE | C<->T | R1529:1H |
| NZ_LS483377.1 | 3824030 | nonsyn | smeE | G<->T | R1528:0S |
| NZ_LS483377.1 | 3824034 | syn | smeE | G<->C | A1524:2A |
| NZ_LS483377.1 | 3824049 | syn | smeE | A<->G | G1509:2G |
| NZ_LS483377.1 | 3824250 | syn | smeE | G<->C | G1308:2G |
| NZ_LS483377.1 | 3824625 | syn | smeE | T<->C | E933:2E |
| NZ_LS483377.1 | 3824627 | nonsyn | smeE | C<->G | E931:0Q |
| NZ_LS483377.1 | 3824655 | syn | smeE | A<->G | D903:2D |
| NZ_LS483377.1 | 3824781 | syn | smeE | C<->T | E777:2E |
| NZ_LS483377.1 | 3824931 | syn | smeE | T<->C | A627:2A |
| NZ_LS483377.1 | 3824937 | nonsyn | smeE | G<->C | I621:2M |
| NZ_LS483377.1 | 3824939 | nonsyn | smeE | T<->C | I619:0V |
| NZ_LS483377.1 | 3824955 | syn | smeE | C<->G | A603:2A |
| NZ_LS483377.1 | 3824970 | syn | smeE | A<->G | Y588:2Y |
| NZ_LS483377.1 | 3824976 | syn | smeE | A<->G | H582:2H |
| NZ_LS483377.1 | 3825063 | syn | smeE | G<->A | R495:2R |
| NZ_LS483377.1 | 3825066 | syn | smeE | A<->G | D492:2D |
| NZ_LS483377.1 | 3825075 | syn | smeE | A<->G | N483:2N |
| NZ_LS483377.1 | 3825120 | syn | smeE | G<->A | N438:2N |
| NZ_LS483377.1 | 3825411 | syn | smeE | G<->A | Y147:2Y |
| NZ_LS483377.1 | 3825522 | syn | smeE | C<->T | A36:2A |
| NZ_LS483377.1 | 3825525 | syn | smeE | G<->A | F33:2F |
| NZ_LS483377.1 | 3825690 | syn | smeD | G<->A | I1065:2I |
| NZ_LS483377.1 | 3825720 | nonsyn | smeD | C<->A | E1035:2D |
| NZ_LS483377.1 | 3825790 | nonsyn | smeD | C<->T | G965:1D |
| NZ_LS483377.1 | 3825804 | syn | smeD | G<->C | T951:2T |
| NZ_LS483377.1 | 3825825 | syn | smeD | G<->A | R930:2R |
| NZ_LS483377.1 | 3825921 | syn | smeD | C<->G | P834:2P |
| NZ_LS483377.1 | 3825978 | syn | smeD | T<->C | E777:2E |
| NZ_LS483377.1 | 3825984 | syn | smeD | G<->A | F771:2F |
| NZ_LS483377.1 | 3826074 | syn | smeD | A<->G | A681:2A |
| NZ_LS483377.1 | 3826086 | syn | smeD | G<->A | R669:2R |
| NZ_LS483377.1 | 3826125 | syn | smeD | G<->C | V630:2V |
| NZ_LS483377.1 | 3826161 | syn | smeD | T<->C | A594:2A |
| NZ_LS483377.1 | 3826164 | syn | smeD | A<->G | N591:2N |
| NZ_LS483377.1 | 3826248 | syn | smeD | A<->G | Y507:2Y |
| NZ_LS483377.1 | 3826285 | nonsyn | smeD | C<->T | R470:1K |
| NZ_LS483377.1 | 3826398 | syn | smeD | G<->C | R357:2R |
| NZ_LS483377.1 | 3826401 | syn | smeD | C<->G | A354:2A |
| NZ_LS483377.1 | 3826413 | syn | smeD | C<->G | T342:2T |
| NZ_LS483377.1 | 3826447 | nonsyn | smeD | C<->T | S308:1N |
| NZ_LS483377.1 | 3826476 | nonsyn | smeD | C<->A | E279:2D |
| NZ_LS483377.1 | 3826481 | nonsyn | smeD | T<->G | I274:0L |
| NZ_LS483377.1 | 3826497 | syn | smeD | G<->A | G258:2G |
| NZ_LS483377.1 | 3826518 | syn | smeD | T<->C | E237:2E |
| NZ_LS483377.1 | 3826548 | syn | smeD | G<->A | N207:2N |
| NZ_LS483377.1 | 3826572 | syn | smeD | G<->C | V183:2V |
| NZ_LS483377.1 | 3826584 | syn | smeD | G<->A | N171:2N |
| NZ_LS483377.1 | 3826596 | syn | smeD | C<->G | P159:2P |
| NZ_LS483377.1 | 3826665 | syn | smeD | C<->T | E90:2E |
| NZ_LS483377.1 | 3826689 | syn | smeD | G<->A | C66:2C |
| NZ_LS483377.1 | 3826703 | nonsyn | smeD | C<->T | A52:0T |
| NZ_LS483377.1 | 3826704 | syn | smeD | T<->C | A51:2A |
| NZ_LS483377.1 | 3826725 | syn | smeD | T<->C | A30:2A |
| NZ_LS483377.1 | 3826728 | syn | smeD | A<->G | F27:2F |
| NZ_LS483377.1 | 4235599 | syn | smeR | G<->C | T9:2T |
| NZ_LS483377.1 | 4235602 | syn | smeR | G<->C | S12:2S |
| NZ_LS483377.1 | 4235612 | nonsyn | smeR | T<->C | S22:0P |
| NZ_LS483377.1 | 4235623 | syn | smeR | C<->T | I33:2I |
| NZ_LS483377.1 | 4235626 | syn | smeR | G<->C | L36:2L |
| NZ_LS483377.1 | 4235632 | syn | smeR | C<->T | V42:2V |
| NZ_LS483377.1 | 4235641 | syn | smeR | G<->A | E51:2E |
| NZ_LS483377.1 | 4235644 | syn | smeR | A<->G | P54:2P |
| NZ_LS483377.1 | 4235654 | nonsyn | smeR | T<->G | S64:0A |
| NZ_LS483377.1 | 4235656 | syn | smeR | G<->C | S66:2S |
| NZ_LS483377.1 | 4235659 | syn | smeR | A<->G | V69:2V |
| NZ_LS483377.1 | 4235680 | syn | smeR | C<->G | A90:2A |
| NZ_LS483377.1 | 4235683 | syn | smeR | C<->G | A93:2A |
| NZ_LS483377.1 | 4235691 | nonsyn | smeR | C<->T | A101:1V |
| NZ_LS483377.1 | 4235698 | syn | smeR | G<->A | E108:2E |
| NZ_LS483377.1 | 4235707 | syn | smeR | C<->T | D117:2D |
| NZ_LS483377.1 | 4235710 | syn | smeR | C<->T | D120:2D |
| NZ_LS483377.1 | 4235713 | syn | smeR | T<->C | G123:2G |
| NZ_LS483377.1 | 4235717 | nonsyn | smeR | C<->A | Q127:0K |
| NZ_LS483377.1 | 4235728 | nonsyn | smeR | C<->G | D138:2E |
| NZ_LS483377.1 | 4235752 | syn | smeR | C<->T | D162:2D |
| NZ_LS483377.1 | 4235788 | syn | smeR | C<->T | D198:2D |
| NZ_LS483377.1 | 4235804 | nonsyn | smeR | C<->A | R214:0S |
| NZ_LS483377.1 | 4235809 | syn | smeR | A<->G | E219:2E |
| NZ_LS483377.1 | 4235815 | syn | smeR | T<->C | R225:2R |
| NZ_LS483377.1 | 4235827 | syn | smeR | T<->C | D237:2D |
| NZ_LS483377.1 | 4235830 | syn | smeR | A<->G | V240:2V |
| NZ_LS483377.1 | 4235836 | syn | smeR | C<->G | V246:2V |
| NZ_LS483377.1 | 4235851 | syn | smeR | A<->G | A261:2A |
| NZ_LS483377.1 | 4235860 | syn | smeR | A<->G | E270:2E |
| NZ_LS483377.1 | 4235938 | syn | smeR | C<->G | V348:2V |
| NZ_LS483377.1 | 4235944 | syn | smeR | G<->A | R354:2R |
| NZ_LS483377.1 | 4235947 | syn | smeR | A<->G | V357:2V |
| NZ_LS483377.1 | 4235948 | nonsyn | smeR | A<->G | M358:0V |
| NZ_LS483377.1 | 4235969 | nonsyn | smeR | C<->A | R379:0S |
| NZ_LS483377.1 | 4235971 | syn | smeR | C<->G | R381:2R |
| NZ_LS483377.1 | 4235982 | nonsyn | smeR | G<->C | G392:1A |
| NZ_LS483377.1 | 4235983 | syn | smeR | T<->C | G393:2G |
| NZ_LS483377.1 | 4235990 | nonsyn | smeR | G<->A | A400:0T |
| NZ_LS483377.1 | 4235996 | nonsyn | smeR | G<->A | G406:0S |
| NZ_LS483377.1 | 4235998 | syn | smeR | T<->C | G408:2G |
| NZ_LS483377.1 | 4236016 | syn | smeR | G<->A | E426:2E |
| NZ_LS483377.1 | 4236025 | syn | smeR | A<->G | A435:2A |
| NZ_LS483377.1 | 4236028 | syn | smeR | C<->T | R438:2R |
| NZ_LS483377.1 | 4236061 | syn | smeR | G<->C | T471:2T |
| NZ_LS483377.1 | 4236088 | syn | smeR | G<->C | T498:2T |
| NZ_LS483377.1 | 4236100 | syn | smeR | C<->G | T510:2T |
| NZ_LS483377.1 | 4236103 | syn | smeR | A<->C | P513:2P |
| NZ_LS483377.1 | 4236118 | syn | smeR | G<->A | A528:2A |
| NZ_LS483377.1 | 4236133 | syn | smeR | C<->T | L543:2L |
| NZ_LS483377.1 | 4236139 | syn | smeR | G<->C | R549:2R |
| NZ_LS483377.1 | 4236142 | syn | smeR | G<->C | L552:2L |
| NZ_LS483377.1 | 4236145 | syn | smeR | C<->T | Y555:2Y |
| NZ_LS483377.1 | 4236154 | syn | smeR | T<->C | H564:2H |
| NZ_LS483377.1 | 4236217 | syn | smeR | C<->T | D627:2D |
| NZ_LS483377.1 | 4236244 | syn | smeR | T<->A | R654:2R |
| NZ_LS483377.1 | 4236256 | syn | smeR | C<->G | G666:2G |
| NZ_LS483377.1 | 4236262 | syn | smeR | C<->T | G672:2G |
